# RNase T2 deficiency promotes TLR13-dependent replenishment of tissue-protective Kupffer cells

**DOI:** 10.1084/jem.20230647

**Published:** 2025-01-24

**Authors:** Ryota Sato, Kaiwen Liu, Takuma Shibata, Katsuaki Hoshino, Kiyoshi Yamaguchi, Toru Miyazaki, Ryosuke Hiranuma, Ryutaro Fukui, Yuji Motoi, Yuri Fukuda-Ohta, Yun Zhang, Tatjana Reuter, Yuko Ishida, Toshikazu Kondo, Tomoki Chiba, Hiroshi Asahara, Masato Taoka, Yoshio Yamauchi, Toshiaki Isobe, Tsuneyasu Kaisho, Yoichi Furukawa, Eicke Latz, Kohta Nakatani, Yoshihiro Izumi, Yunzhong Nie, Hideki Taniguchi, Kensuke Miyake

**Affiliations:** 1 Division of Innate Immunity, The Institute of Medical Science, The University of Tokyo, Minato-ku, Japan; 2 Department of Immunology, Faculty of Medicine, Kagawa University, Miki, Japan; 3 Laboratory for Inflammatory Regulation, RIKEN Center for Integrative Medical Science (IMS-RCAI), Yokohama, Japan; 4 Division of Clinical Genome Research, The Institute of Medical Science, The University of Tokyo, Minato-ku, Japan; 5 The Institute for AIM Medicine, Tokyo, Japan; 6 Department of Immunology, Institute of Advanced Medicine, Wakayama Medical University, Kimiidera, Japan; 7 Institute of Innate Immunity, University Hospital Bonn, University of Bonn, Bonn, Germany; 8 Department of Forensic Medicine, Wakayama Medical University, Kimiidera, Japan; 9 Department of Systems Biomedicine, Tokyo Medical and Dental University, Bunkyo-ku, Japan; 10 Department of Molecular and Experimental Medicine, The Scripps Research Institute, La Jolla, CA, USA; 11 Department of Chemistry, Graduate School of Science, Tokyo Metropolitan University, Tokyo, Japan; 12 Deutsches Rheuma Forschungszentrum Berlin (DRFZ), Berlin, Germany; 13 Division of Metabolomics, Medical Institute of Bioregulation, Kyushu University, Higashi-ku, Japan; 14 Division of Regenerative Medicine, Center for Stem Cell Biology and Regenerative Medicine, The Institute of Medical Science, The University of Tokyo, Minato-ku, Japan

## Abstract

Lysosomal stress due to the accumulation of nucleic acids (NAs) activates endosomal TLRs in macrophages. Here, we show that lysosomal RNA stress, caused by the lack of RNase T2, induces macrophage accumulation in multiple organs such as the spleen and liver through TLR13 activation by microbiota-derived ribosomal RNAs. TLR13 triggered emergency myelopoiesis, increasing the number of myeloid progenitors in the bone marrow and spleen. Splenic macrophages continued to proliferate and mature into macrophages expressing the anti-inflammatory cytokine IL-10. In the liver, TLR13 activated monocytes/macrophages to proliferate and mature into monocyte-derived KCs (moKCs), in which, the liver X receptor (LXR) was activated. In accumulated moKCs, tissue clearance genes such as MerTK, AXL, and apoptosis inhibitor of macrophage (AIM) were highly expressed, while TLR-dependent production of proinflammatory cytokines was impaired. Consequently, *Rnaset2*^−/−^ mice were resistant to acute liver injuries elicited by acetaminophen (APAP) and LPS with D-galactosamine. These findings suggest that TLR13 activated by lysosomal RNA stress promotes the replenishment of tissue-protective Kupffer cells.

## Teaser

Sato et al. report that the lack of RNase T2 activates TLR13, the sensor of bacterial 23S ribosomal RNAs, leading to the accumulation of tissue-protective Kupffer cells. Consequently, *Rnaset2*^*−/−*^ mice are resistant against acute liver injury caused by acetaminophen.

## Graphical Abstract

**Figure:**
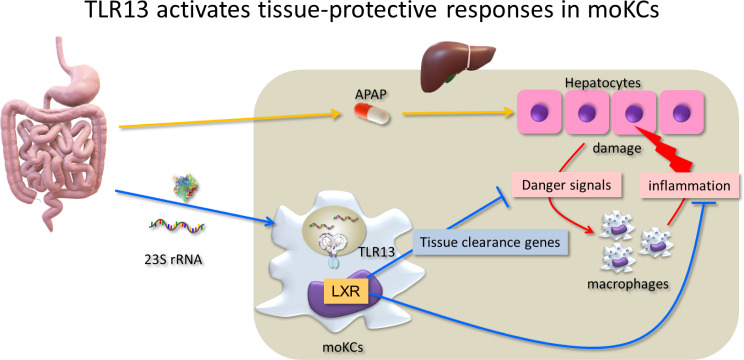

## Introduction

Extracellular RNA serves as an immune signal, alerting macrophages to pathogen invasion and tissue damage (Miyake et al., 2017; Roers et al., 2016). These RNAs are internalized by macrophages and degraded in the endosomal compartment, where RNA-sensing Toll-like receptors (TLRs), such as TLR3, TLR7, TLR8, and TLR13, are located (Marshak-Rothstein and Rifkin, 2007; Miyake et al., 2017, 2018). TLR3 is specific for double-stranded RNAs (dsRNAs), while TLR13 detects single-stranded RNAs (ssRNAs) from bacterial ribosomal RNA (rRNA) (Li and Chen, 2012; Oldenburg et al., 2012). TLR7 and TLR8 recognize RNA degradation products, which are combinations of oligoribonucleotides and nucleosides (Shibata et al., 2016; Tanji et al., 2015; Zhang et al., 2016, 2018). Notably, TLR8 and 13 work only in humans and mice, respectively.

Lysosomal nucleosides are transported to the cytoplasm via the transporter SLC29A3. Mutations in this transporter cause histiocytic diseases, collectively known as SLC29A3 disorders (Cliffe et al., 2009; Molho-Pessach et al., 2008). When SLC29A3 function is impaired, nucleosides accumulate in lysosomes, a condition we term lysosomal nucleoside stress. This stress activates TLR7 and TLR8, promoting the survival and proliferation of macrophages in mice and humans, respectively (Hsu et al., 2012; Shibata et al., 2023; Shiloh et al., 2023).

Lysosomal RNA stress occurs when RNA degradation in lysosomes is compromised. RNase T2, an endosomal enzyme, is the sole member of the T2 family of RNases and functions at acidic pH to degrade RNAs (Luhtala and Parker, 2010). Loss-of-function mutations in the RNase T2 gene lead to cystic leukoencephalopathy without megalencephaly in humans (Henneke et al., 2009; Rice et al., 2017), characterized by bilateral anterior temporal subcortical cysts and multifocal white matter lesions, along with psychomotor impairment, spasticity, and epilepsy (Tonduti et al., 2016). Consistently, *Rnaset2*^−/−^ mice exhibit type I IFN–dependent neuroinflammation (Kettwig et al., 2021). RNase T2 negatively regulates TLR3 responses by degrading dsRNA (Liu et al., 2021) but positively regulates mouse TLR7 and human TLR8 by producing their ligands (Greulich et al., 2019; Liu et al., 2021; Ostendorf et al., 2020). In *Slc29a3*^−/−^ mice, lysosomal nucleoside stress activates TLR7, leading to splenomegaly, whereas lysosomal RNA stress in *Rnaset2*^−/−^ mice results in both hepatomegaly and splenomegaly (Kettwig et al., 2021), suggesting that RNA stress activate distinct responses compared with nucleoside stress.

The liver, exposed to blood from the gut, frequently encounters intestinal bacteria and drugs entering the portal system. Kupffer cells (KCs), hepatic resident macrophages clearing bacteria, aged neutrophils, and senescent platelets (Ginhoux and Guilliams, 2016; Shi et al., 2001), have dual roles in liver injuries, such as in acetaminophen (APAP) overdose-induced acute liver injury. KCs can either mitigate or exacerbate tissue damage by clearing or responding to danger signals from damaged hepatocytes (Imaeda et al., 2009; Ishida et al., 2006; Ju et al., 2002). Although KCs originate from embryonic precursors (Li et al., 2022), their deletion prompts monocyte differentiation into monocyte-derived KCs (moKCs), guided by niche-derived signals such as the Notch ligand DLL4, TFG-β/BMPs, and desmosterol, an LXR ligand (Bonnardel et al., 2019; Guilliams et al., 2013). LXR ligands, produced by sinusoidal endothelial cells and hepatocytes, maintain the KC pool at steady state. During infections, TLRs drive the replenishment of peripheral macrophages through emergency myelopoiesis (Jackson et al., 2023), but their role in KC replenishment remains unclear.

In this study, we investigated *Rnaset2*^−/−^ mice and discovered that the bacterial rRNA sensor TLR13 facilitated macrophage accumulation in the spleen and liver. This accumulation was reduced by antibiotic treatment, indicating TLR13 activation by microbiota-derived rRNAs. TLR13 stimulated splenic macrophages to proliferate and secrete IL-10. In the liver, TLR13 induced monocytes/macrophages to mature into moKCs, where the transcription factors LXR and MafB were activated. These accumulated moKCs exhibited a reduced inflammatory response to TLR ligands and elevated expression of tissue-clearance molecules such as AIM, C1qb, Axl, and MerTK. Consequently, *Rnaset2*^−/−^ mice showed resistance to acute liver injuries caused by APAP or LPS + D-galactosamine challenge. These findings suggest that lysosomal RNA stress due to RNase T2 deficiency activates TLR13-dependent replenishment of tissue-protective moKCs.

## Results

### TLR13 drives splenomegaly and hepatomegaly in *Rnaset2*^−/−^ mice

**Figure S1.**
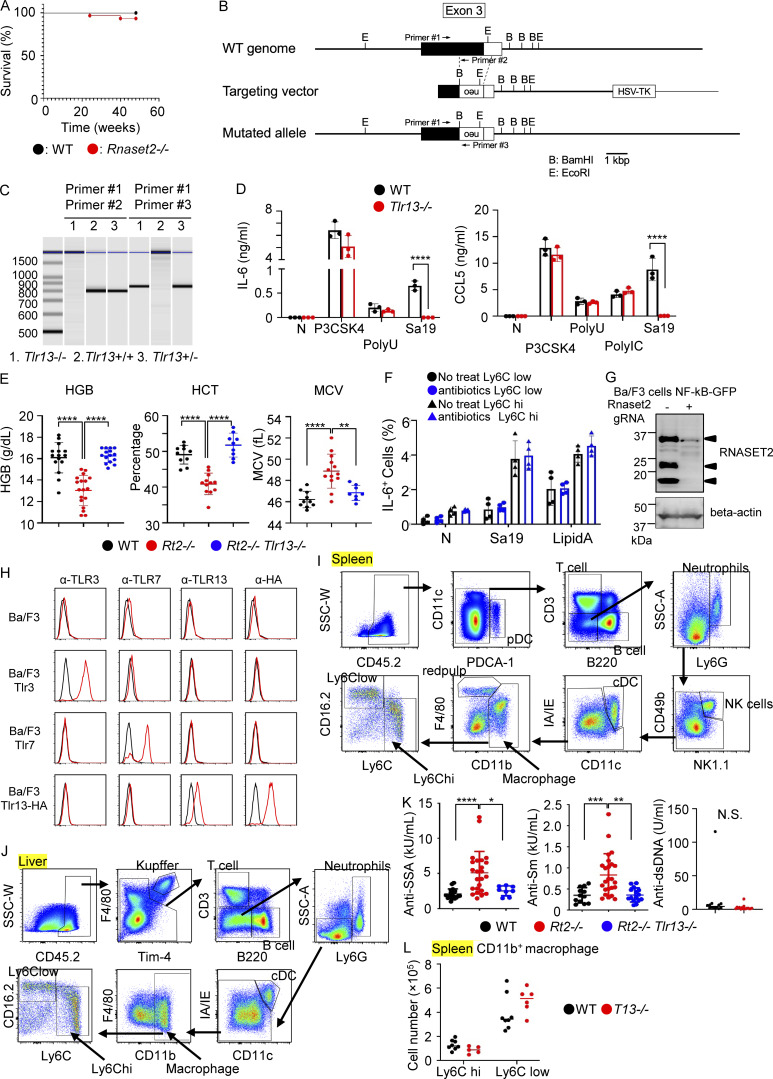
**Analyses of *Tlr13*** ^**−/−**^ **, *Rnaset2*** ^**−/−**^ **, and *Rnaset2*** ^**−/−**^ ***Tlr13*** ^**−/−**^ **mice. (A)** Survival curve of wild-type mice (black) and *Rnaset2*^*−/−*^ mice (red). **(B)** Schematic representation of *Tlr13* gene targeting. The filled and open boxes represent coding and 3′-untranslated regions of the *Tlr13* gene, respectively. Neo indicates the neomycin resistance gene: B, BamH I; and E, EcoR I. **(C)** PCR analyses with primers indicated in B to determine genotypes of indicated mice. **(D)** Production of IL-6 and CCL5 by BM-derived macrophages left unstimulated or stimulated with indicated TLR ligands. Results represent mean values with SD from triplicates. **(E)** Hb concentration, hematocrit, and mean corpuscular volume of RBCs in indicated mice (*n* = 8–16). **(F)** Percentage of IL-6^+^ cells in splenic Ly6C^low^ or Ly6C^hi^ macrophages from wild-type mice with or without antibiotics treatment after in vitro stimulation with the TLR13 ligand Sa19 (5 µg/ml) or the TLR4 ligand lipid A (1 µg/ml) together with Brefeldin A for 3 h (*n* = 3). **(G)** Immunoblotting of whole cell lysates from wild-type and *Rnaset2*^−/−^ Ba/F3 with anti-RNase T2 mAb. β-actin was also immunoprobed to show that the amounts of samples are equal. **(H)** Ba/F3 expressing Tlr3, Tlr7, or Tlr13-HA tag were subjected to membrane-permeabilized staining with mAbs to TLR3 (PaT3), TLR7 (A94B10), TLR13 (M13), and αHA. **(I and J)** The gating strategy of splenic cells (I) and hepatic cells (J). **(K)** Serum titer of autoantibodies to SSA, Sm, and dsDNAs in indicated mice (*n* = 8–26). **(L)** The numbers of CD11b^+^ Ly6C^hi^ and CD11b^+^ Ly6C^low^ macrophages in the spleens of indicated mice (*n* = 5–8). *P < 0.05, **P < 0.01, ***P < 0.001 and ****P < 0.0001. Source data are available for this figure: SourceData FS1.

**Figure 1.**
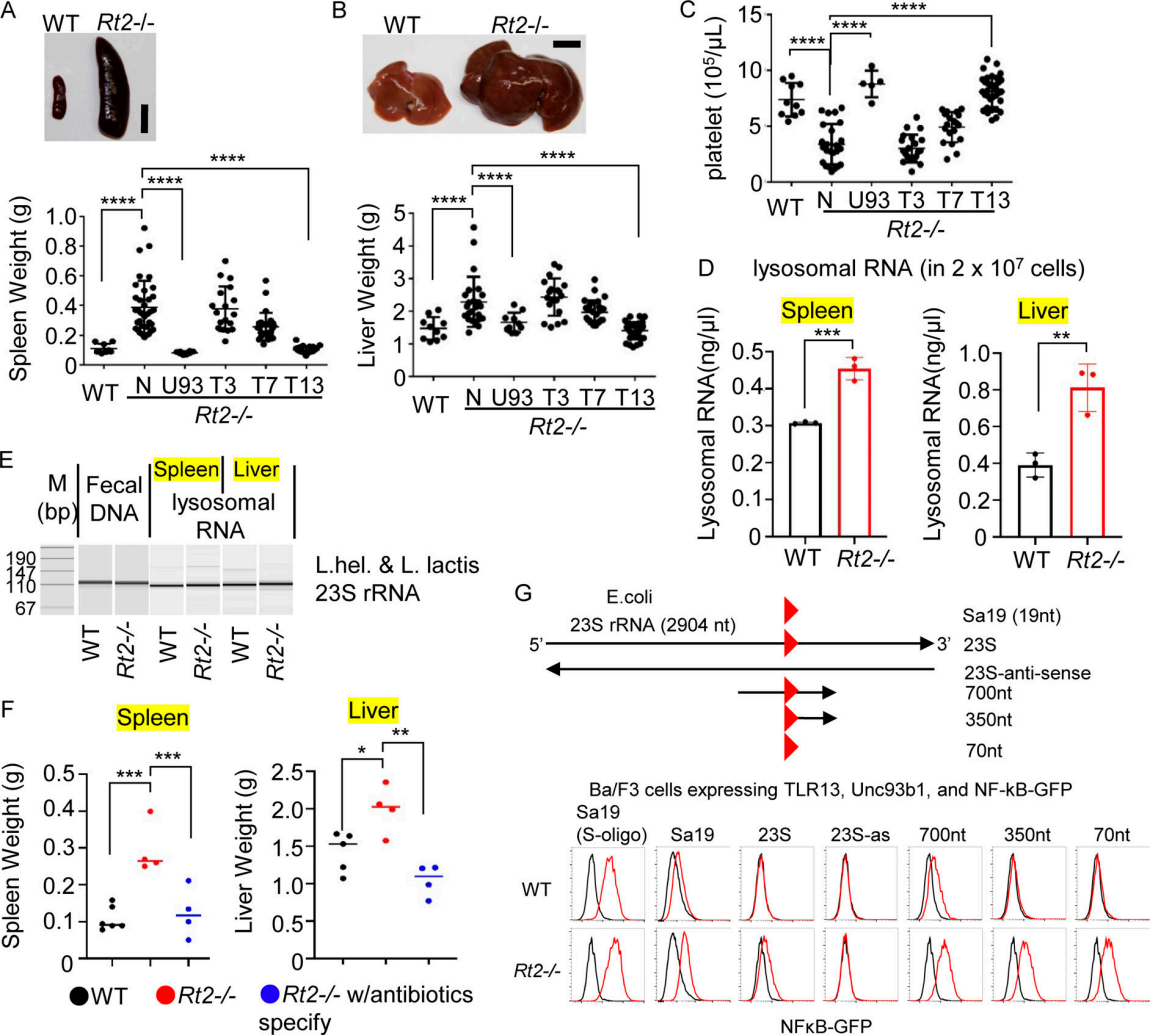
**TLR13 responses to bacterial rRNAs drive hepatosplenomegaly. (A)** Spleen photograph and splenic weights of wild-type (WT), *Rt2*^−/−^ (*Rnaset2*^−/−^), *Rt2*^−/−^*Unc93b1*^−/−^ (U93), *Rt2*^−/−^*Tlr3*^−/−^ (T3), *Rt2*^−/−^*Tlr7*^−/−^ (T7), and *Rt2*^−/−^*Tlr13*^−/−^ (T13) mice (*n* = 8–25). **(B)** Liver photograph and liver weights of indicated mice (*n* = 10–25). **(C)** Platelet counts of indicated mice (*n* = 4–25). **(D)** RNA concentrations of lysosomal fractions from spleen and liver of wild-type and *Rt2*^−/−^ mice. **(E)** PCR amplification of 23S rRNA of *Lactobacillus helveticus* and *Lactococcus lactis*. PCR templates were fecal DNA and cDNAs prepared from lysosomal RNAs from D. **(F)** Dot plots show weights of spleen and liver from wild-type and *Rt2*^−/−^ mice with or without antibiotic treatment (*n* = 4–6). **(G)** Schematic diagram of fragments derived from *E. coli* 23S rRNA. The red triangle denotes the minimal 12-mer sequence to activate TLR13. Ba/F3 cells expressing TLR13, Unc93b1, and NFκB-GFP were stimulated with modified Sa19 (S-oligo), Sa19, and rRNA fragments of indicated length at 25 µg/ml. Red and gray histograms show GFP expression in wild-type and *Rt2*^−/−^ Ba/F3 cells left unstimulated (black) and stimulated with indicated ssRNA for 24 h (red). *P < 0.05, **P < 0.01, ***P < 0.001 and ****P < 0.0001. Source data are available for this figure: SourceData F1.

Mouse RNase T2 is encoded by two genes, *Rnaset2a* and *Rnaset2b*. To study the role of RNase T2 in vivo, we previously generated *Rnaset2a*^−/−^*Rnaset2b*^−/−^ mice (Liu et al., 2021), hereafter described as *Rnaset2*^−/−^ or *Rt2*^−/−^ mice. These mice were born at Mendelian ratios and developed normally. Despite the report of premature death in *Rnaset2*^−/−^ mice (Kettwig et al., 2021), the mice in our animal facility did not exhibit early mortality (Fig. S1 A). However, they did develop splenomegaly, hepatomegaly, and thrombocytopenia, consistent with previous findings (Fig. 1, A–C) (Kettwig et al., 2021). RNase T2 is localized to endosomes (Liu et al., 2021); thus, its deficiency leads to RNA accumulation within these compartments (Haud et al., 2011; Huang et al., 2015). We hypothesized that the phenotypes observed in *Rnaset2*^−/−^ mice result from the activation of endosomal RNA-sensing TLRs. Given that Unc93b1 is essential for the function of all RNA-sensing TLRs (Tabeta et al., 2006), we generated *Rnaset2*^−/−^*Unc93b1*^−/−^ mice. These mice did not develop splenomegaly, hepatomegaly, and thrombocytopenia (Fig. 1, A–C), supporting our hypothesis. To further delineate the role of RNA-sensing TLRs, we crossed *Rnaset2*^−/−^ mice with *Tlr3*^−/−^, *Tlr7*^−/−^, and the newly established *Tlr13*^−/−^ mice (Fig. S1, B–D). We found that the phenotypes observed in *Rnaset2*^−/−^ mice were not present in *Rnaset2*^−/−^*Tlr13*^−/−^ mice (Fig. 1, A–C). Additionally, consistent with the previous report (Kettwig et al., 2021), *Rnaset2*^−/−^ mice exhibited macrocytic anemia, which was also dependent on TLR13 (Fig. S1 E). These results suggest that TLR13 is a critical driver of the most phenotypes observed in *Rnaset2*^−/−^ mice.

### TLR13 is activated by bacterial rRNA from the microbiome

Consistent with the previous findings in RNase T2–deficient zebrafish (Haud et al., 2011), we observed that the amounts of RNA recovered from lysosomal fractions of the spleen and liver in *Rnaset2*^−/−^ mice were ∼1.5- and 2-fold larger, respectively, than those in wild-type mice (Fig. 1 D). PCR analyses further revealed that lysosomal RNAs in both wild-type and *Rnaset2*^−/−^ mice included the Lactobacillus 23S rRNA-derived sequence capable of activating TLR13 (Fig. 1 E). Fecal DNA also contained the sequence of the TLR13 ligand (Fig. 1 E), suggesting that bacterial rRNAs from the gut reach the liver and spleen. To investigate the role of the microbiome in TLR13-dependent phenotypes, *Rnaset2*^−/−^ mice were treated with a cocktail of antibiotics, namely metronidazole, neomycin, ampicillin, and vancomycin, from 3 to 6 wk of age. This treatment did not impair IL-6 production by Ly6C^hi^ and Ly6C^low^ macrophages upon stimulation with the TLR13 ligand Sa19 or lipid A (Fig. S1 F), but ameliorated splenomegaly and hepatomegaly in *Rnaset2*^−/−^ mice (Fig. 1 F).

To further understand the role of RNase T2 in TLR13 activation, we used the IL-3–dependent Ba/F3 cell line, which was transduced to express TLR13, Unc93b1, and NF-κB-GFP. In this cell line, the *Rnaset2* genes were deleted using RNase T2-targeting gRNA and Cas9 (Fig. S1 G). The cells were then stimulated with 23S rRNAs of various lengths (Fig. 1 G), and GFP expression was evaluated by FACS analyses. RNase T2-deficient Ba/F3 cells responded comparably with wild-type Ba/F3 cells to RNase-resistant TLR13 ligand Sa19 but exhibited a heightened response to RNase-sensitive Sa19 and ssRNA fragments. Neither wild-type nor RNase T2-deficient Ba/F3 cells responded to anti-sense ssRNA fragment, indicating that their responses were sequence dependent. These findings suggest that RNase T2 negatively regulates TLR13 activation by bacterial rRNAs.

### Accumulation of TLR13-expressing macrophages in the spleen and liver

**Figure 2.**
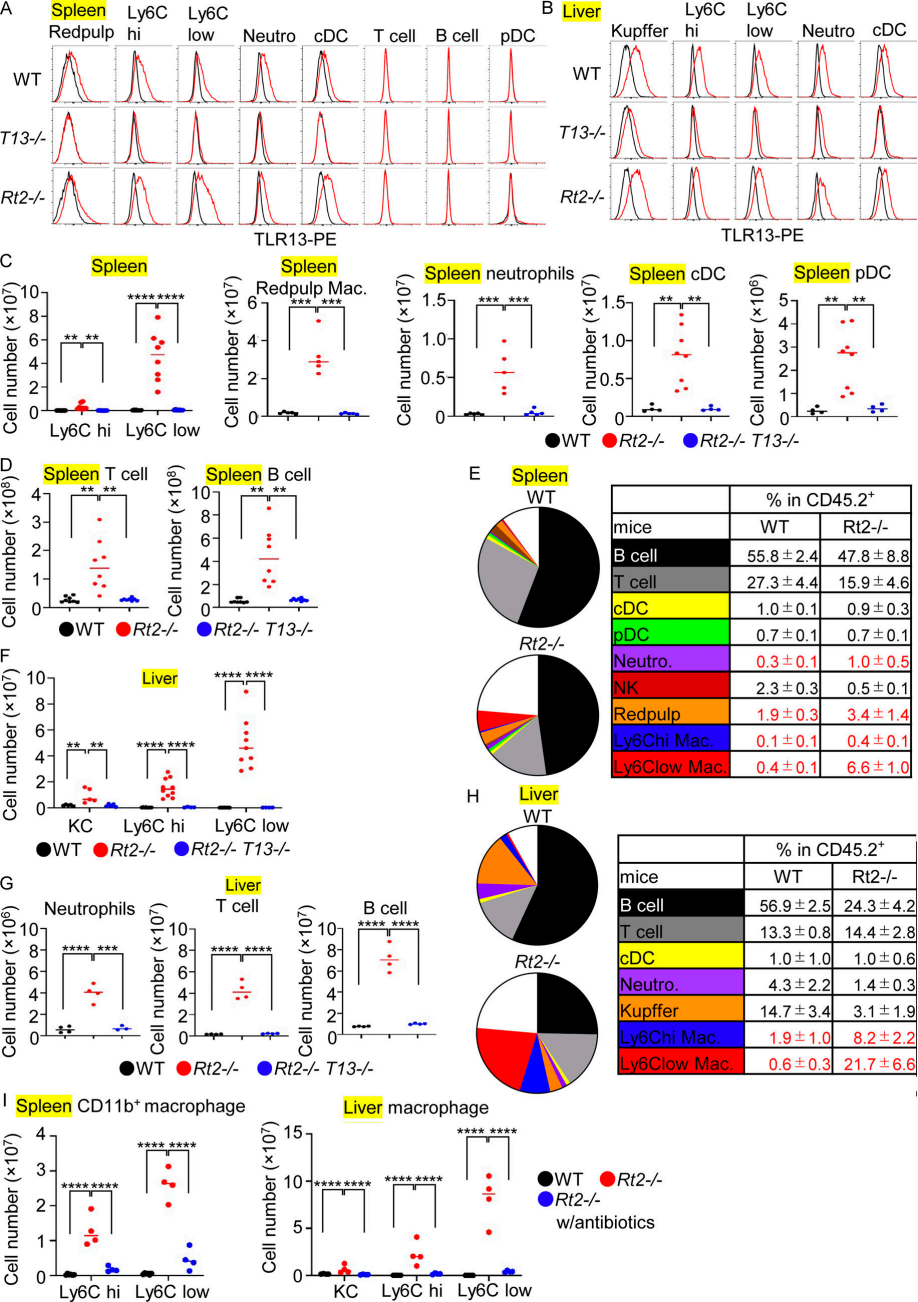
**TLR13-dependent macrophage accumulation in the spleen and liver. (A and B)** TLR13 expression in indicated immune cells in the spleen (A) and liver (B) of indicated mice. Red and black histograms show staining with anti-TLR13 mAb and isotype-matched antibody, respectively. **(C and D)** The cell numbers of indicated immune cells in the spleen from indicated mice (*n* = 5–8). **(E and H)** The percentages of indicated immune cells in the spleen and liver of wild-type and *Rt2*^−/−^ mice. **(F and G)** The cell numbers of indicated immune cells in the liver in indicated mice (*n* = 5–8). **(I)** The cell numbers of splenic or hepatic macrophages in indicated mice with or without antibiotics treatment (*n* = 4). **P < 0.01, ***P < 0.001 and ****P < 0.0001.

We next examined TLR13 expression in immune cells using newly generated anti-TLR13 mAb (Fig. S1 H) and observed that TLR13 protein was expressed in Ly6C^hi^ and Ly6C^low^ macrophages, red pulp macrophages, neutrophils, and conventional dendritic cells (cDCs), but not in T cells, B cells, or plasmacytoid DCs (pDCs) in the spleen (Fig. 2 A and Fig. S1 I). Similarly, myeloid cells such as KCs, Ly6C^hi^, and Ly6C^low^ macrophages, neutrophils, and cDCs expressed TLR13 in the liver (Fig. 2 B and Fig. S1 J).

The numbers of splenic immune cells were all increased in *Rnaset2*^−/−^ mice (Fig. 2, C and D) but only macrophages and neutrophils increased also in percentage (Fig. 2 E). T and B cells did not express TLR13 (Fig. 2 A), but they increased in number (Fig. 2 D), suggesting that splenic lymphocytes were activated by TLR13-expressing macrophages and cDCs. Lymphocyte activation resulted in production of autoantibodies against RNA-associated antigens such as SSA and Sm in *Rnaset2*^−/−^ mice (Fig. S1 K). All the hepatic immune cells increased in number, but only Ly6C^hi^ and Ly6C^low^ macrophages, not KCs, increased also in percentage (Fig. 2, F–H). The increases in these macrophages in the spleen and liver were abolished by antibiotics treatment (Fig. 2 I). Additionally, TLR13 deficiency alone did not alter the numbers of splenic macrophages compared to wild-type mice (Fig. S1 L). These results suggest that TLR13 activation in Ly6C^hi^ and Ly6C^low^ macrophages in the spleen and liver resulted in their accumulation in *Rnaset2*^−/−^ mice.

### TLR13 drives emergency myelopoiesis in the spleen

**Figure S2.**
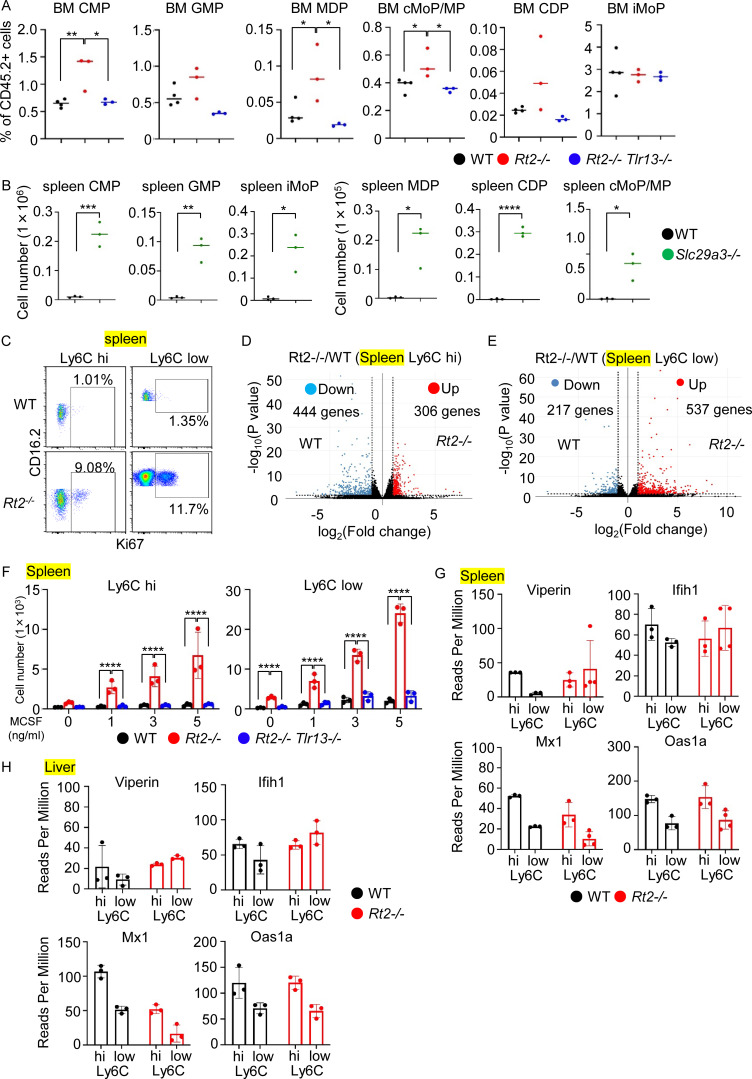
**TLR13 responses in the** **BM** **, spleen, and liver of *Rnaset2*** ^***−/−***^ **mice. (A)** The numbers of monocyte/DC progenitors in the BM of indicated mice (*n* = 3–4). **(B)** The numbers of monocyte/DC progenitors in the spleen of WT and *Slc29a3*^*−/−*^ mice (*n* = 3–4). **(C)** Dot plots show the expression of Ki67 and CD16.2 in splenic macrophages from WT and *Rnaset2*^*−/−*^ mice. **(D and E)** Volcano plots displaying log_2_ fold change of expression (x axes) and log_10_ normalized expression (y axes) for the comparison of splenic Ly6C^hi^ macrophages from *Rnaset2*^*−/−*^ mice versus those from wild-type mice (*n* = 3) (D), and that of splenic Ly6C^low^ macrophages from *Rnaset2*^*−/−*^ mice versus those from wild-type mice (*n* = 3–4) (E). **(F)** Bars show the numbers of Ly6C^hi^ and Ly6C^low^ macrophages from indicated mice that survived in vitro 3 days culture with M-CSF (0, 1, 3, 5 ng/ml). **(G and H)** Bars and dots show reads per million (RPM) of indicated ISGs in indicated splenic and liver macrophages (*n* = 3–4). *P < 0.05, **P < 0.01, ***P < 0.001 and ****P < 0.0001.

**Figure 3.**
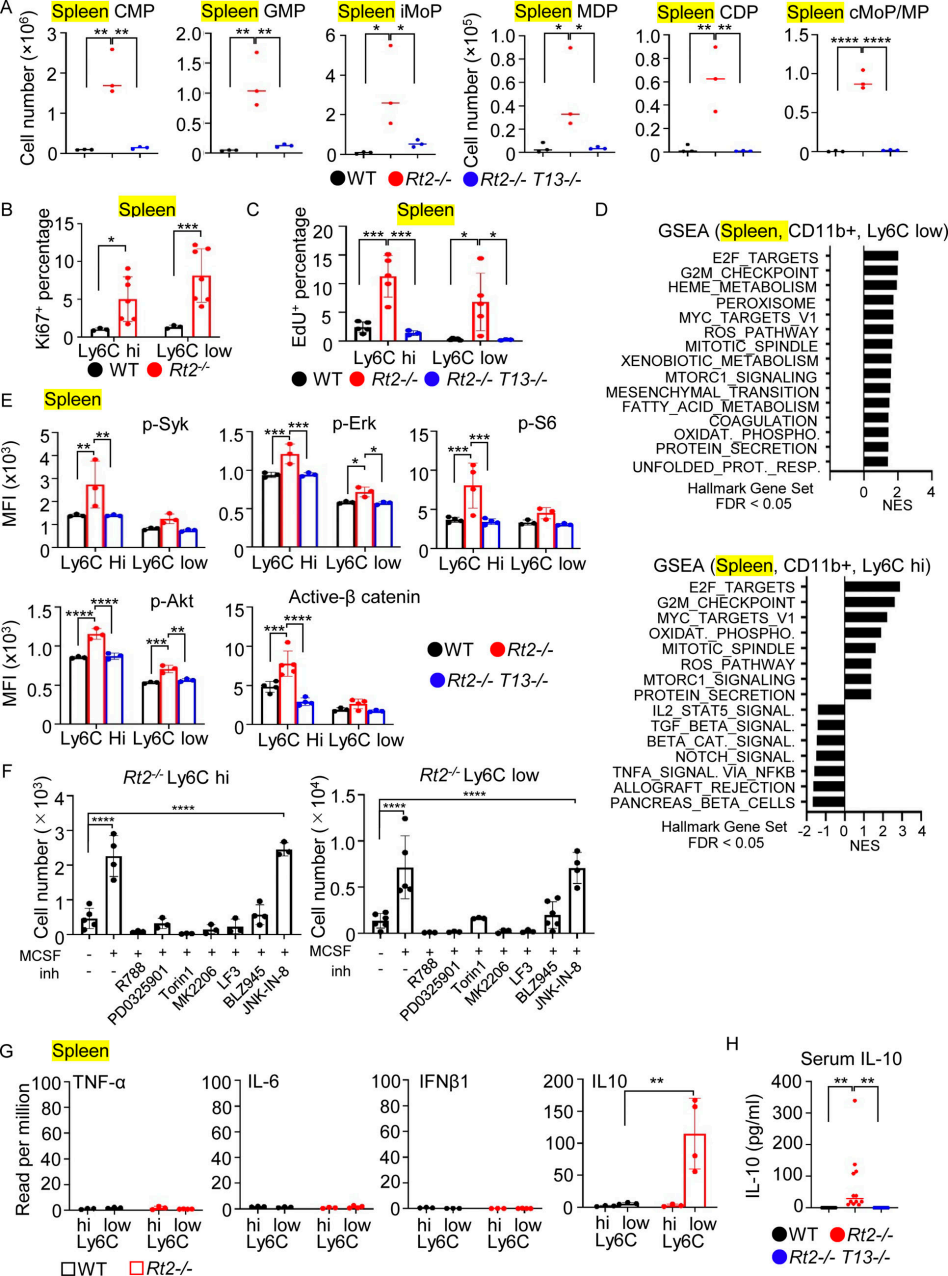
**Emergency myelopoiesis, macrophage proliferation, and Il-10 production in the spleen. (A)** The numbers of monocyte/DC progenitors in the spleen of indicated mice (*n* = 3). **(B and C)** The percentages of Ki67^+^ and EdU^+^ cells in splenic Ly6C^hi^ and Ly6C^low^ macrophages from indicated mice (*n* = 7). **(D)** GSEA of genes with >1.5-fold changes in comparison of splenic Ly6C^hi^ and Ly6C^low^ macrophages from *Rt2*^*−/−*^ mice versus those from wild-type mice. The bars indicate normalized enrichment scores (NESs) of hallmark gene sets with FDR < 0.05. **(E)** Mean fluorescence intensity (MFI) values of flow cytometry staining of splenic Ly6C^hi^ and Ly6C^low^ macrophages from indicated mice with antibodies to indicated signaling molecules (*n* = 3–5). **(F)** Bars show the numbers of splenic Ly6C^hi^ and Ly6C^low^ macrophages from *Rt2*^−/−^ mice that survived in vitro 3 days culture with M-CSF (5 ng/ml) and indicated inhibitors such as Syk (R788, 1 µM), MEK (PD0325901, 1 µM), mTOR (Torin 1, 0.25 µM), AKT (MK2266, 1 µM), β-catenin (LF3, 30 µM), and CSF1R (BLZ945, 1 µM), or JNK (JNK-IN-8, 1 µM). **(G)** Bars and dots show reads per million (RPM) of TNF-α, IL-6, IFNβ1, and IL10 in indicated splenic macrophages (*n* = 3–4). **(H)** Serum levels of IL-10 in indicated mice (*n* = 12–14). *P < 0.05, **P < 0.01, ***P < 0.001 and ****P < 0.0001.

Given the consistent increases in the numbers and percentages of macrophages in the spleen of *Rnaset2*^−/−^ mice, we focused on cellular mechanisms underlying TLR13-dependent increases in splenic macrophages. TLR ligands enhance monocyte production through extramedullary myelopoiesis, a process termed emergency myelopoiesis (Burberry et al., 2014; Chavakis et al., 2019; Nagai et al., 2006; Ueda et al., 2005). To explore the role of emergency myelopoiesis on the increase in splenic macrophages, we examined the numbers of monocyte/DC progenitors in the bone marrow (BM) and spleen of *Rnaset2*^−/−^ mice. In the BM, there was an increase in the percentages of common myeloid progenitors (CMPs), monocyte-DC progenitors (MDPs), and common monocyte progenitors/monocyte progenitors (cMoP/MPs) (Fig. S2 A). However, there was no increase in granulocyte-monocyte progenitors (GMPs), common DC progenitors (CDPs), or inducible monocyte progenitors (iMoPs). In the spleen, the numbers of monocyte/DC progenitors such as CMPs, GMPs, MDPs, CDPs, cMoP/MPs, and iMoPs were TLR13 dependently increased (Fig. 3 A). Similar increases of monocyte/DC progenitors in the spleen were observed in *Slc29a3*^−/−^ mice (Fig. S2 B), suggesting that TLR7 also induced emergency myelopoiesis in *Slc29a3*^−/−^ mice. These findings suggest that TLR13 increases the number of macrophages through emergency myelopoiesis in *Rnaset2*^−/−^ mice.

### TLR13 activates splenic macrophages to proliferate and produce IL-10

To investigate whether TLR13 continues to drive the proliferation of monocytes/macrophages, we examined the expression of the proliferation-associated antigen Ki67 and EdU uptake. We found that both Ly6C^hi^ and Ly6C^low^ macrophages from *Rnaset2*^−/−^ mice showed increases in Ki67 expression and the uptake of the thymidine analogue EdU (Fig. 3, B and C; and Fig. S2 C). Transcriptome analyses were conducted to further characterize TLR13 responses in splenic macrophages. In Ly6C^hi^ macrophages from *Rnaset2*^−/−^ mice, 306 genes were upregulated and 444 genes were downregulated by >1.5-fold compared with Ly6C^hi^ macrophages from wild-type mice (Fig. S2 D). Additionally, 614 genes were upregulated, while 498 genes were downregulated, with a q value of <0.1 (Data S1). In Ly6C^low^ macrophages from *Rnaset2*^−/−^ mice, 537 genes were upregulated and 217 genes were downregulated by >1.5-fold (Fig. S2 E). Furthermore, 382 genes were upregulated and 250 genes were downregulated with a q value of <0.1 (Data S1). Gene set enrichment analysis (GSEA) revealed that proliferation-associated hallmarks such as “E2F targets,” “G2M checkpoint,” “MYC targets V1,” “mitotic spindle,” and “mTORC1 signaling” were positively enriched in differentially expressed genes (DEGs) of both Ly6C^hi^ and Ly6C^low^ splenic macrophages from *Rnaset2*^−/−^ mice compared with wild-type mice (Fig. 3 D). These findings suggest that splenic macrophages continue to proliferate in a manner dependent on TLR13 in *Rnaset2*^−/−^ mice.

We next examined the TLR13 signaling pathways activated in splenic macrophages using FACS analyses. We observed TLR13-dependent increases in phosphorylation of Syk, Erk, S6, and AKT, as well as in the activated form of β-catenin in Ly6C^hi^ macrophages (Fig. 3 E). In Ly6C^low^ macrophages, TLR13-dependent increases in phosphorylation of Erk and Akt were also noted. To determine whether these signaling pathways drive macrophage proliferation, we cultured splenic macrophages in the presence of M-CSF at 5 ng/ml and added various inhibitors. This setup allowed proliferation only in macrophages from *Rnaset2*^−/−^ mice, not in wild-type macrophages (Fig. S2 F). The survival of Ly6C^hi^ and Ly6C^low^ macrophages from *Rnaset2*^−/−^ mice in culture was downregulated with inhibitors for Syk (R788), MEK (PD0325901), mTOR (Torin 1), AKT (MK2266), β-catenin (LF3), and CSF1R (BLZ945), but not with JNK inhibitor (JNK-IN-8) (Fig. 3 F). Although FACS analyses did not detect the activated form of β-catenin and phosphorylation of Syk and S6 in Ly6C^low^ macrophages from *Rnaset2*^−/−^ mice (Fig. 3 E), the effect of inhibitors suggested their activation. These results indicate that TLR13 in splenic Ly6C^hi^ and Ly6C^low^ macrophages from *Rnaset2*^−/−^ mice activates signaling pathways mediated by Syk, Erk, mTOR, and β-catenin to drive proliferation.

Although both Ly6C^hi^ and Ly6C^low^ macrophages proliferated in the spleen of *Rnaset2*^−/−^ mice, the latter predominantly accumulated over the former (Fig. 2 C). Ly6C^hi^ macrophages were likely to mature into Ly6C^low^ macrophages in the spleen of *Rnaset2*^−/−^ mice (Ginhoux and Jung, 2014). In Ly6C^low^ macrophages, transcriptome analyses showed that the hallmark “TNFA signal via NFKB” was negatively enriched (Fig. 3 D), suggesting the lack of inflammatory responses. Consistently, expression of mRNAs encoding proinflammatory cytokines such as TNF-α and IL-6, IFNβ1, and IFN-stimulated genes (ISGs) were not increased (Fig. 3 G and Fig. S2 G), whereas the mRNA for the anti-inflammatory cytokine IL-10 was highly expressed in splenic Ly6C^low^ macrophages from *Rnaset2*^−/−^ mice. Additionally, serum IL-10 levels in *Rnaset2*^−/−^ mice were TLR13-dependently increased (Fig. 3 H), suggesting that TLR13 activation promotes macrophage maturation into IL-10–producing macrophages in the spleen.

### TLR13 drives proliferation and maturation of hepatic macrophage

**Figure 4.**
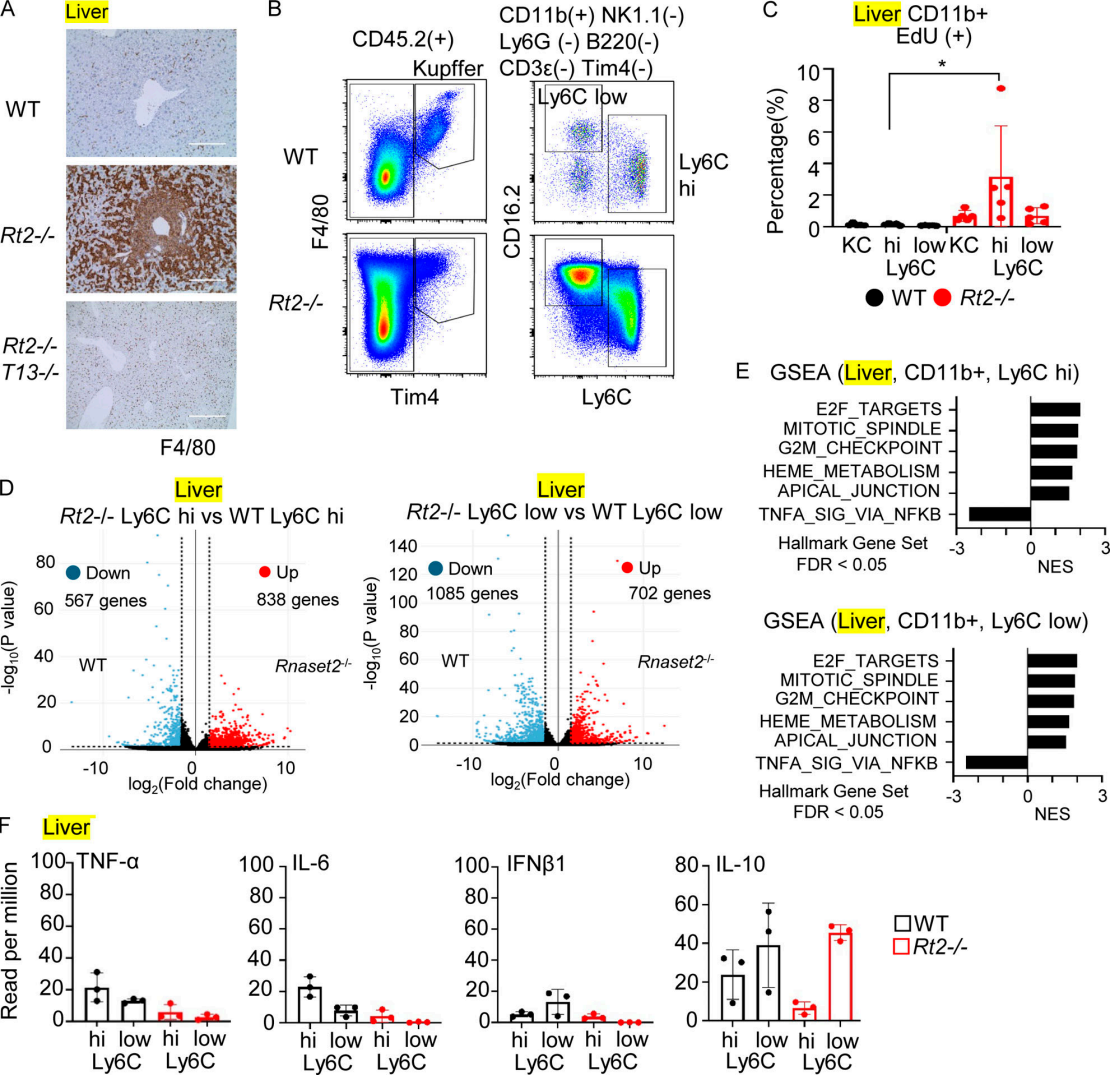
**TLR13-dependent macrophage proliferation in the liver. (A)** Immunohistochemistry showing F4/80 expression in the livers of indicated mice. Scale bar, 200 μm. **(B)** FACS dot plots show expression of F4/80, Tim4, Ly6C, and CD16.2 in hepatic macrophages from indicated mice. **(C)** The percentages of the EdU^+^ cells in indicated hepatic macrophage subsets from indicated mice. **(D)** Volcano plots show log_2_ fold change of expression (x axes) and log_10_ normalized expression (y axes) for the comparisons of hepatic Ly6C^hi^ and Ly6C^low^ macrophages from *Rnaset2*^−/−^ mice versus those from wild-type mice (*n* = 3). Genes with >1.5-fold upregulated and downregulated expression are shown in red and blue, respectively. **(E)** GSEA of genes with >1.5-fold changes in comparison of hepatic Ly6C^hi^ and Ly6C^low^ macrophages from Rnaset2^−/−^ mice versus those from wild-type mice. The bars indicate normalized enrichment scores (NESs) of hallmark gene sets with FDR < 0.05. **(F)** Bars and dots show reads per million (RPM) of TNF-α, IL-6, IFNβ1, and IL10 in indicated splenic macrophages (*n* = 3). *P < 0.05.

We next focused on hepatic immune cells in *Rnaset2*^−/−^ mice (Fig. 4, A and B). Although immune cells including macrophages, neutrophils, cDCs, and lymphocytes increased in number (Fig. 2, F and G), only Ly6C^hi^ and Ly6C^low^ macrophages increased also in percentage (Fig. 2 H), suggesting that these macrophages predominantly increased in the liver of *Rnaset2*^−/−^ mice. Because EdU uptake into nuclear DNAs was TLR13-dependently increased only in hepatic Ly6C^hi^ macrophages (Fig. 4 C), TLR13 was suggested to drive proliferation of Ly6C^hi^ macrophages and their maturation into Ly6C^low^ macrophages. To further understand TLR13 responses, we conducted transcriptome analyses of hepatic macrophages. In hepatic Ly6C^hi^ macrophages from *Rnaset2*^−/−^ mice, 838 genes were upregulated and 567 genes were downregulated by >1.5-fold compared with those from wild-type mice (Fig. 4 D). In addition, 835 genes were upregulated and 1,202 genes were downregulated with a q value of <0.1 in Ly6C^hi^ macrophages from *Rnaset2*^−/−^ mice (Data S1). In hepatic Ly6C^low^ macrophages from *Rnaset2*^−/−^ mice, 702 genes were upregulated and 1,085 genes were downregulated by >1.5-fold compared with those from wild-type mice (Fig. 4 D). Also, 1,245 genes were upregulated and 1,519 genes were downregulated with a q value of <0.1 (Data S1). GSEA demonstrated that proliferation-associated gene sets such as “E2F targets,” “mitotic spindle,” and “G2M checkpoint” were positively enriched in both Ly6C^hi^ and Ly6C^low^ hepatic macrophages from *Rnaset2*^−/−^ mice (Fig. 4 E). Although we did not detect significant EdU uptake by Ly6C^low^ macrophages (Fig. 4 C), it is possible that a population of hepatic Ly6C^low^ macrophages that have just matured from Ly6C^hi^ macrophages still proliferated.

In hepatic Ly6C^low^ macrophages, there were no increases in IL-10 mRNA as well as in mRNAs for proinflammatory cytokines such as TNF-α, IL-6, IFNβ1, and ISGs (Fig. 4 F and Fig. S2 H), indicating that TLR13 responses in hepatic macrophages differ from those in splenic macrophages.

### TLR13-dependent macrophage accumulation in the brain, lung, and kidney

**Figure S3.**
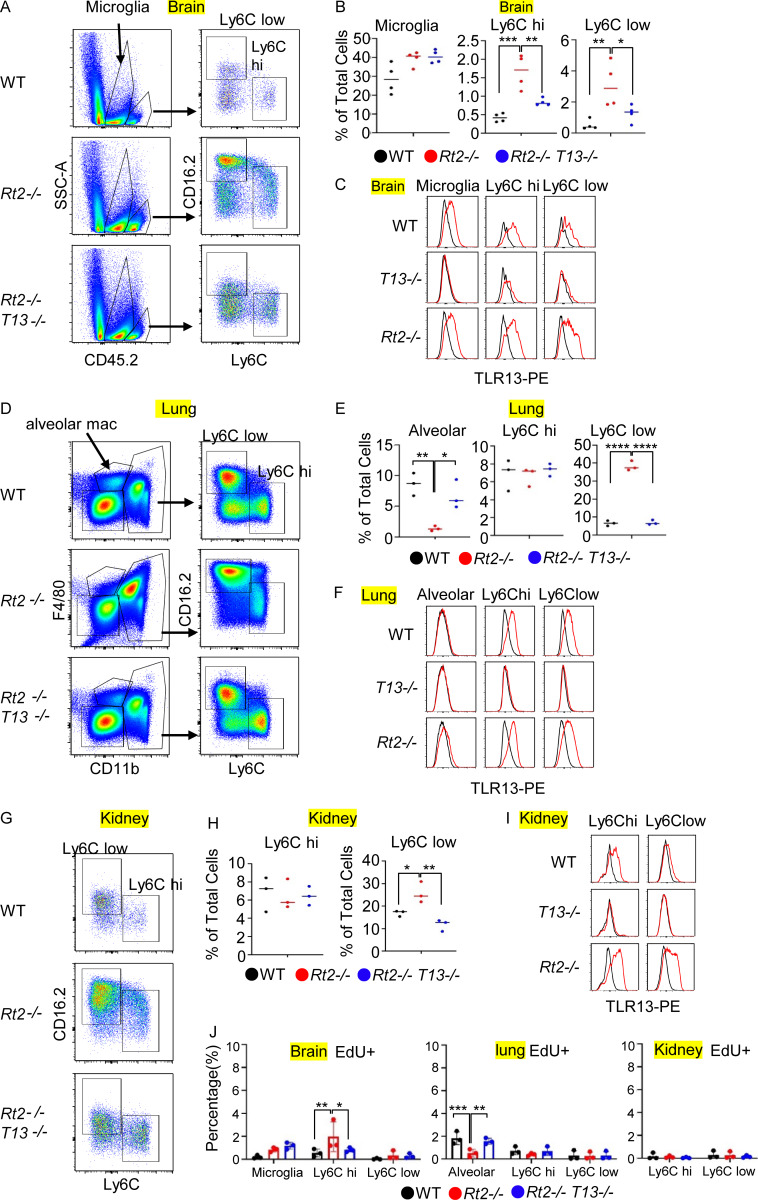
**TLR13 responses in various organs of *Rnaset2*** ^***−/−***^ **. (A)** Gating strategy to detect microglia, Ly6C^hi^, and Ly6C^low^ macrophages in the brains of indicated mice. **(B)** The percentages of F4/80^+^ CD45.2^int^ microglia, Ly6C^hi^, and Ly6C^lo^ macrophage in CD45^+^ cells from the brain of indicated mice (*n* = 4). **(C, F, and I)** TLR13 expression in indicated immune cells in the brain, lung, and kidney of indicated mice. Red and black histograms show staining with anti-TLR13 mAb and isotype-matched antibody, respectively. **(D)** Gating strategy to detect alveolar, Ly6C^hi,^ and Ly6C^low^ macrophages from the lungs of indicated mice. **(E)** The percentages of F4/80^+^ CD11b^int^ alveolar, Ly6C^hi^, and Ly6C^low^ macrophage in CD45^+^ cells from the lungs of indicated mice (*n* = 4). **(G)** Gating strategy to detect Ly6C^hi^ and Ly6C^low^ macrophages from the kidneys of indicated mice. **(H)** The percentages of Ly6C^hi^ and Ly6C^low^ macrophages in CD45^+^ cells from the kidney of indicated mice (*n* = 3). **(J)** The percentages of the EdU^+^ cells in indicated macrophage subsets from the indicated organs of indicated mice. *P < 0.05, **P < 0.01, ***P < 0.001 and ****P < 0.0001.

In addition to the spleen and liver, we investigated macrophages in the brain, lung, and kidney. In the brain, both Ly6C^hi^ and Ly6C^low^ macrophages TLR13-dependently increased (Fig. S3, A and B). Microglia, similar to Ly6C^hi^ and Ly6C^low^ macrophages, expressed TLR13 (Fig. S3 C) but did not significantly increase in percentage (Fig. S3 B). In the lung and kidney, only Ly6C^low^ macrophages TLR13-dependently increased in percentage (Fig. S3, D, E, G, and H), although both Ly6C^hi^ and Ly6C^low^ macrophages expressed TLR13 (Fig. S3, F and I). EdU uptake by these macrophages showed that only Ly6C^hi^ macrophages in the brain proliferate (Fig. S3 J), suggesting that bacterial rRNA might enter the brain. In the lung and kidney of *Rnaset2*^−/−^ mice, although Ly6C^low^ macrophages increased, they did not show any increase in EdU uptake compared with those in wild-type mice, suggesting that TLR13 prolongs survival of Ly6C^low^ macrophages in the lung and kidney of *Rnaset2*^−/−^ mice. In contrast to monocyte-derived macrophages (moMAs), alveolar macrophages decreased in *Rnaset2*^−/−^ mice due to TLR13-dependent inhibition of their proliferation (Fig. S3, E and J). These findings demonstrate that TLR13 activation results in macrophage accumulation in multiple organs.

### TLR13-dependent LXR activation in hepatic macrophages

**Figure 5.**
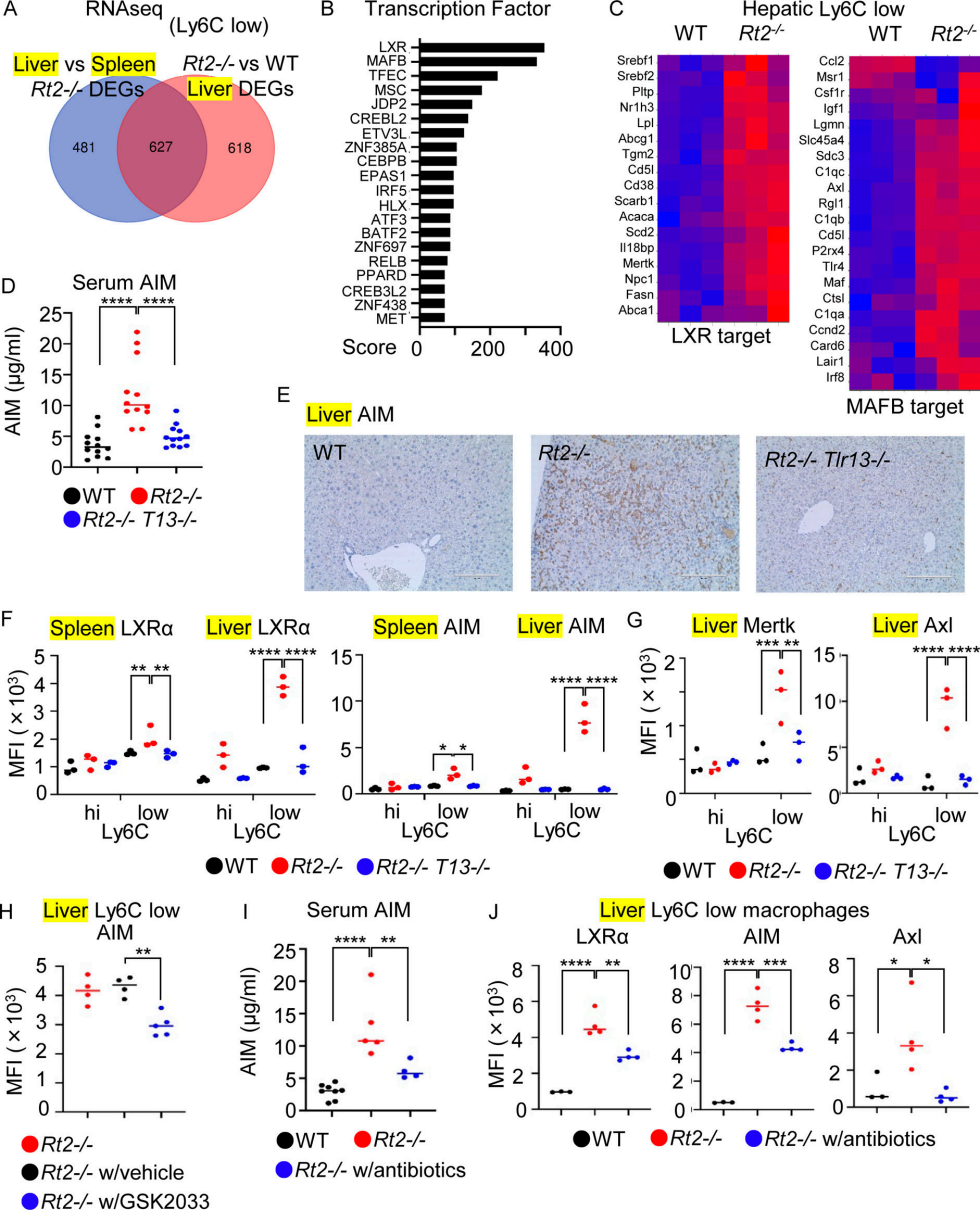
**LXRα activation in hepatic Ly6C** ^**low**^ **macrophages of *Rnaset2*** ^***−/−***^ **mice. (A)** Venn diagrams show the numbers of DEGs in the comparisons of hepatic versus splenic Ly6C^low^ macrophages from *Rt2*^*−/−*^ mice (blue) and of hepatic Ly6C^low^ macrophages from *Rt2*^*−/−*^ mice versus those from wild-type mice. **(B)** Enrichr analyses of the 627 DEGs identified by the above two comparisons (A). **(C)** Heatmaps showing expression of LXR and MAFB target genes in hepatic Ly6C^low^ macrophages from indicated mice. **(D)** Serum levels of AIM in indicated mice (*n* = 12). **(E)** Immunohistochemistry of AIM expression in the liver of indicated mice. Scale bar, 200 µm. **(F and G)** Dot plots show the MFI values of indicated proteins in splenic and hepatic Ly6C^low^ macrophages from indicated mice. **(H)** MFI values of AIM in hepatic Ly6C^low^ macrophages with or without treatment of LXR inhibitor GSK 2033 (*n* = 4–5). **(I and J)** Serum levels of AIM and MFI values of LXRα, AIM and Axl of hepatic Ly6C^low^ macrophages in indicated mice with or without antibiotics (*n* = 5–8 for I, *n* = 3–4 for J). *P < 0.05, **P < 0.01, ***P < 0.001 and ****P < 0.0001.

**Figure S4.**
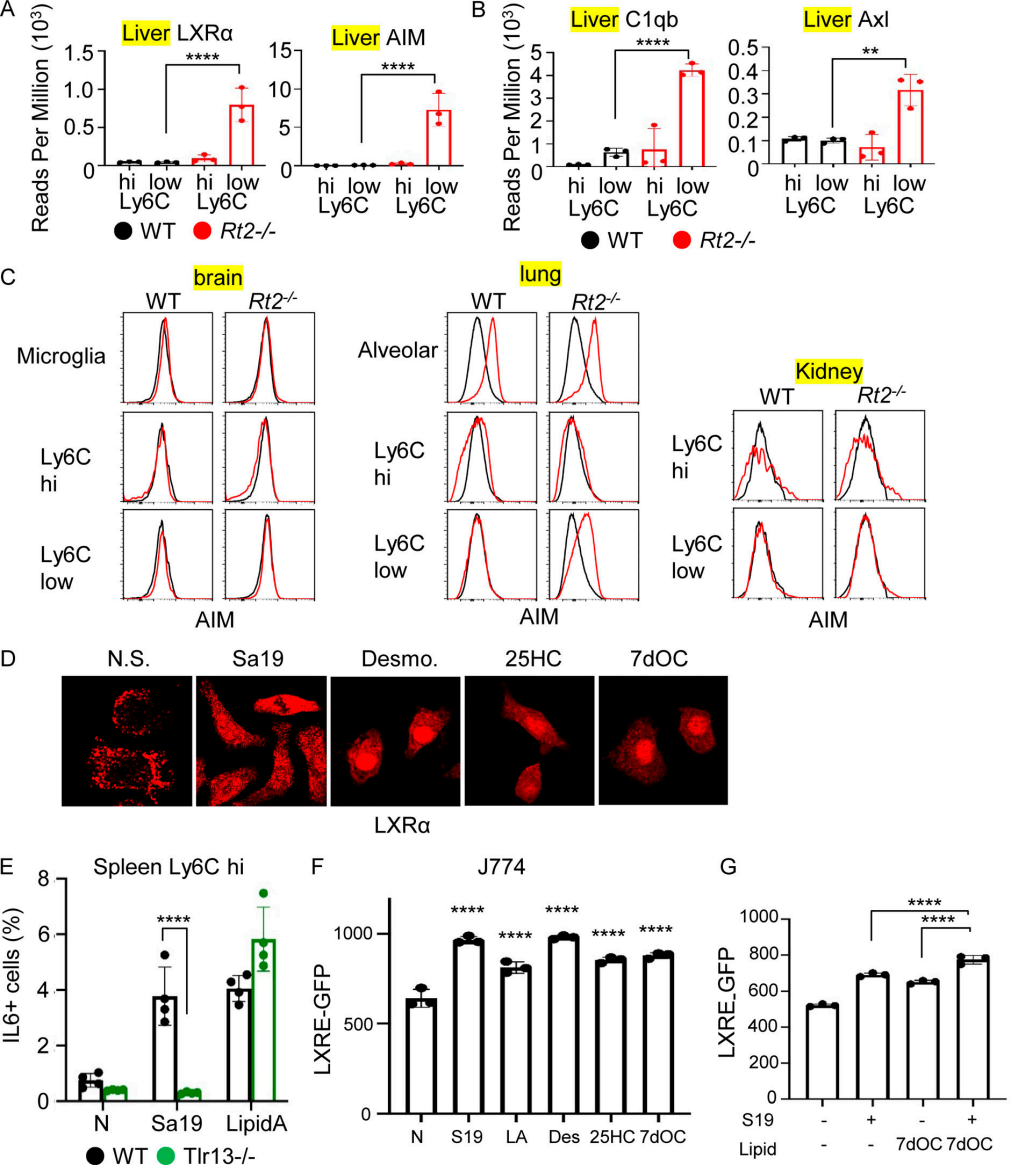
**TLR13 activates transcription factor LXRα in macrophages. (A and B)** Bars show reads per million (RPM) of LXRα, AIM (A), C1qb, and Axl (B) in Ly6C^hi^ and Ly6C^low^ macrophages from wild-type and *Rnaset2*^*−/−*^ mice. **(C)** Histograms show staining with anti-AIM (red) and isotype-matched antibodies (black) in macrophages from the brain, lung, and kidney. **(D)** Localization of LXR in J774 cells left unstimulated or stimulated with the TLR13 ligand (Sa19) or LXR ligands such as Desmosterol, 25HC, and 7dOC. **(E)** The percentages of IL-6^+^ cells in splenic Ly6C^hi^ macrophages from indicated mice after in vitro stimulation with the TLR13 ligand Sa19 (5 µg/ml) or the TLR4 ligand lipid A (1 µg/ml) together with Brefeldin A for 3 h. **(F and G)** MFI values of of GFP in LXRE-GFP-expressing J774 cells after stimulation with Sa19 (5 µg/ml), lipid A (1 µg/ml) and LXR agonists (10 µg/ml). **P < 0.01 and ****P < 0.0001.

We next focused on TLR13 responses in hepatic Ly6C^low^ macrophages from *Rnaset2*^−/−^ mice because Ly6C^low^ macrophages predominantly accumulated in both the spleen and liver and expression of IL-10 mRNA was increased in splenic Ly6C^low^ macrophages but not hepatic Ly6C^low^ macrophages (Fig. 2, C and F; Fig. 3 G; and Fig. 4 F). To characterize TLR13 responses, we compared the gene expression profiles of splenic and hepatic Ly6C^low^ macrophages. In hepatic Ly6C^low^ macrophages of *Rnaset2*^−/−^ mice, 627 genes showed increased expression in two comparisons: *Rnaset2*^−/−^ versus wild-type Ly6C^low^ macrophages in the liver; and hepatic versus splenic Ly6C^low^ macrophages in *Rnaset2*^−/−^ mice (Fig. 5 A). Analyses of these 627 genes using the “Enrichr” search engine with the “All RNA-seq and ChIP-seq sample and signature search 4 (ARCHS4)” database suggested activation of transcription factors such as LXR and MafB (Fig. 5 B) (Kuleshov et al., 2016). The target genes of LXRα and MafB were more highly expressed in hepatic Ly6C^low^ macrophages from *Rnaset2*^−/−^ mice compared with wild-type mice (Fig. 5 C). For instance, LXRα target genes such as LXRα itself and AIM (encoded by *Cd5l*), as well as MafB target genes such as C1qb and Axl, were upregulated in Ly6C^low^ macrophages from *Rnaset2*^−/−^ mice (Fig. S4, A and B) (Hamada et al., 2014; Rébé et al., 2009; Sato et al., 2018; Tran et al., 2017). AIM protein levels in the circulation and liver increased in a TLR13-dependent manner (Fig. 5, D and E). FACS analyses showed that expression levels of LXRα and AIM were more strongly upregulated in Ly6C^low^ macrophages from the liver than from the spleen (Fig. 5 F). Although AIM is a secretory protein, we detected AIM protein within macrophages by membrane-permeabilized staining. Additionally, MerTK and AXL were also TLR13-dependently increased in hepatic Ly6C^low^ macrophages from *Rnaset2*^−/−^ mice (Fig. 5 G). AIM protein expression in hepatic Ly6C^low^ macrophages was decreased by administration of the LXRα antagonist GSK2033 (Fig. 5 H). Furthermore, antibiotic treatment significantly reduced serum levels of AIM and mean fluorescence intensity (MFI) values of LXRα, AIM, and Axl in Ly6C^low^ macrophages (Fig. 5, I and J). These results suggest that TLR13 activates LXR rather than inducing IL-10 production in hepatic Ly6C^low^ macrophages.

To investigate LXR activation in macrophages of the brain, lung, and kidney in *Rnaset2*^−/−^ mice, we analyzed AIM expression using FACS analyses. We found that only lung Ly6C^low^ macrophages from *Rnaset2*^−/−^ mice expressed AIM (Fig. S4 C).

To directly study TLR13-dependent LXR activation, we used the J774 macrophage line, which constitutively expressed LXRα (Fig. S4 D), similar to hepatic Ly6C^low^ macrophages in *Rnaset2*^−/−^ mice. J774 cells were transduced with an LXR response element-GFP reporter construct. The TLR13 ligand Sa19, which induced IL-6 production in splenic macrophages from wild-type mice but not *Tlr13*^−/−^ mice (Fig. S4 E), significantly increased LXR-dependent GFP expression to levels comparable with those induced by LXR ligands such as desmosterol, 25-hydroxycholesterol, and 7-dehydrocholesterol (Fig. S4 F). Sa19 also induced LXRα translocation into the nucleus, although translocation was weaker than that induced by LXR ligands (Fig S4 D). Another TLR ligand, lipid A, also increased GFP expression. Additionally, Sa19 enhanced LXR activation by the LXR ligand 7-dehydrocholesterol (Fig. S4 G), suggesting that Sa19 differentially activated LXR from 7dOC. These results suggest that TLR13 activates LXR in hepatic Ly6C^low^ macrophages in *Rnaset2*^−/−^ mice.

### Accumulation of moKCs in *Rnaset2*^−/−^ mice

**Figure 6.**
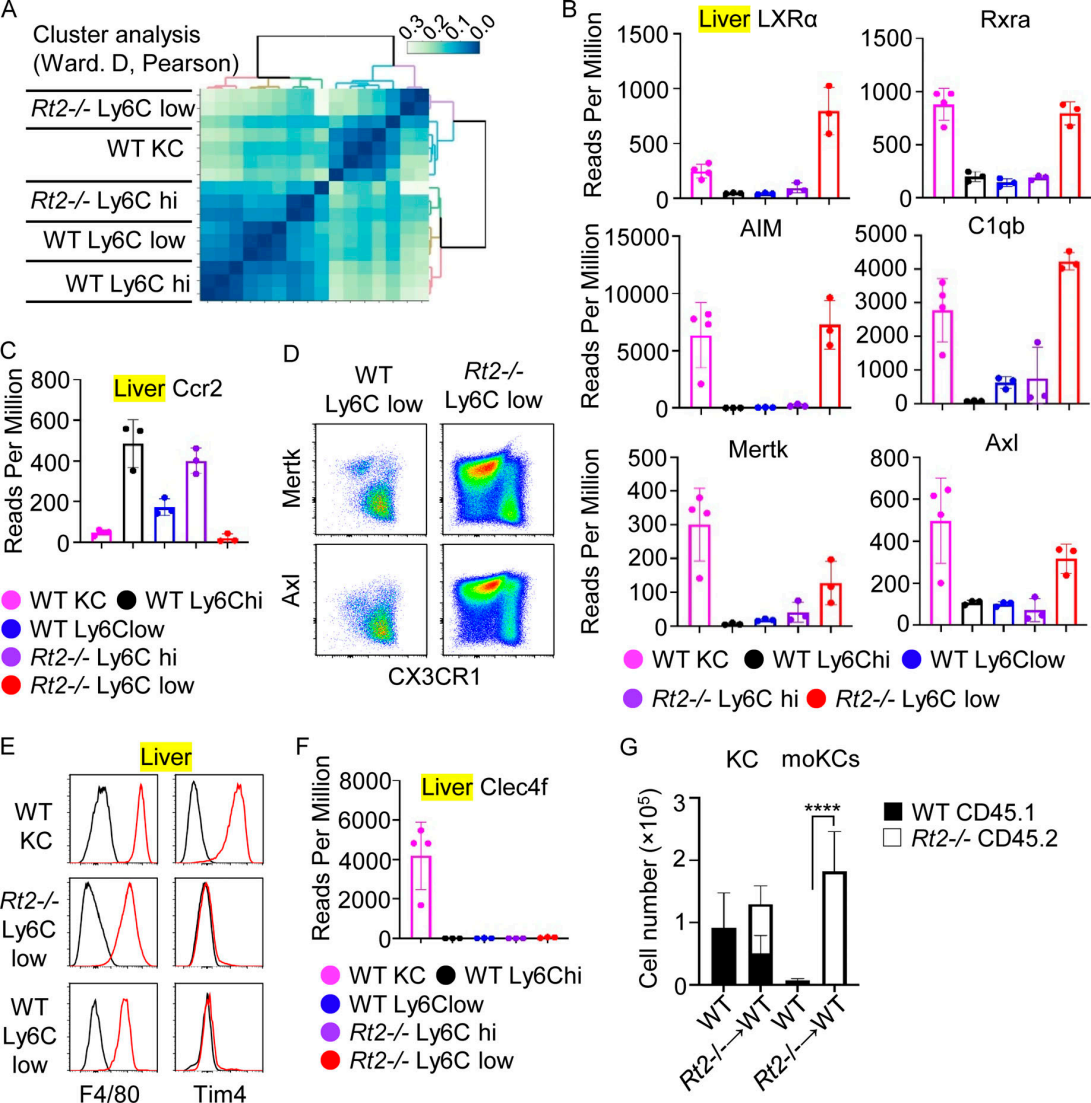
**Hepatic Ly6C** ^**low**^ **macrophages in *Rnaset2*** ^**−/−**^ **mice are moKCs. (A)** Cluster analyses of the genes expressed in Ly6C^hi^ and Ly6C^low^ macrophages from wild-type and *Rnaset2*^−/−^ mice (*n* = 3–4). **(B, C, and F)** Bars show reads per million (RPM) of indicated genes in indicated macrophages (*n* = 3–4). **(D)** Dot plots show expression of MerTK, Axl, and CX3CR1 in hepatic Ly6C^low^ macrophages from wild-type and *Rnaset2*^−/−^ mice. **(E)** Red and black histograms show staining with antibodies to indicated molecules and isotype-matched control, respectively. **(G)** The numbers of KCs and moKCs in wild-type mice and those that were irradiated and received BM cells from *Rnaset2*^−/−^ mice. Closed and open bars show the numbers of macrophages from wild-type (CD45.1) and *Rnaset2*^−/−^ mice (CD45.2). ****P < 0.0001.

LXR is a key transcription factor essential for the initiation and maintenance of KCs (Sakai et al., 2019). Additionally, LXR and MafB are activated in moKCs that replace KCs following their deletion (Sakai et al., 2019). Cluster analyses of hepatic macrophage transcriptomes revealed that hepatic Ly6C^low^ macrophages from *Rnaset2*^−/−^ mice were more similar to wild-type KCs than to Ly6C^hi^ macrophages from *Rnaset2*^−/−^ mice, as well as to Ly6C^hi^ and Ly6C^low^ macrophages from wild-type mice (Fig. 6 A). For instance, mRNAs encoding LXRα, RXRα, AIM, Mertk, Axl, and C1qb were highly expressed, while CCR2 mRNA was poorly expressed in both wild-type KCs and hepatic Ly6C^low^ macrophages from *Rnaset2*^−/−^ mice (Fig. 6, B and C). FACS analyses showed that a major population of Ly6C^low^ macrophages from wild-type mice expressed CX3CR1, but not Mertk or Axl, whereas a major population of Ly6C^low^ macrophages from *Rnaset2*^−/−^ mice highly expressed Mertk and Axl but poorly expressed CX3CR1 (Fig. 6 D). Despite these similarities to KCs, hepatic Ly6C^low^ macrophages from *Rnaset2*^−/−^ mice, like moKCs (Bonnardel et al., 2019; Sakai et al., 2019; Scott et al., 2016; Seidman et al., 2020), did not express the KC markers Tim4 and Clec4f (Fig. 6, E and F). Furthermore, *Rnaset2*^−/−^ BM cells transferred into irradiated wild-type mice gave rise to F4/80^+^ Tim4^−^ Ly6C^low^ macrophages as well as KCs (Fig. 6 G). These results suggest that TLR13 promotes the maturation of monocyte/macrophage into moKCs in *Rnaset2*^−/−^ mice.

### moKCs in *Rnaset2*^−/−^ mice are tissue-protective

**Figure 7.**
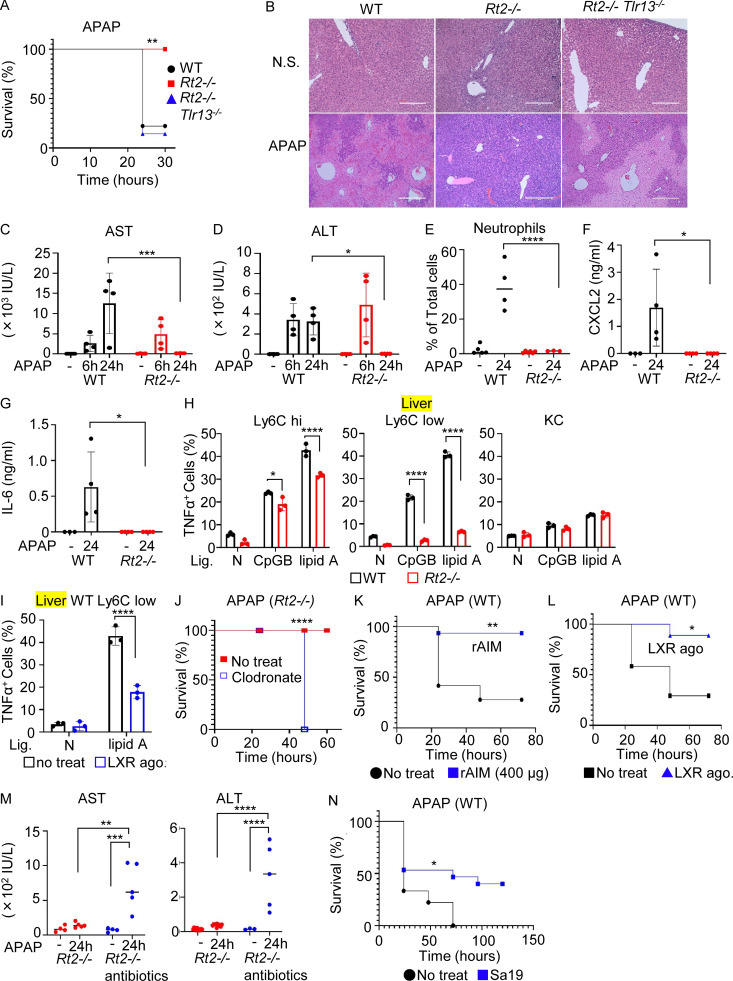
***Rnaset2*** ^**−/−**^ **mice are resistant to acute liver injuries. (A)** Survival curve of indicated mice with APAP challenge at 750 mg/kg (*n* = 11–15). **(B)** H&E staining of the liver of indicated mice 24 h after APAP challenge at 500 mg/kg. Scale bar, 400 µm. **(C, D, F, and G)** Dot plots show serum levels of AST (C), ALT (D), CXCL2 (F), and IL-6 (G) in indicated mice at 6 and 24 h after APAP challenge at 500 mg/kg (*n* = 4–6). **(E)** Dot plots show the percentages of neutrophils that infiltrated the liver in indicated mice 24 h after APAP challenge at 500 mg/kg (*n* = 4). **(H)** Percentage of TNF-α^+^ cells in indicated hepatic macrophage subsets from wild-type and *Rt2*^*−/−*^ mice after in vitro culture with the TLR9 ligand CpG-B (200 nM) or the TLR4/MD-2 ligand lipid A (1 µg/ml) together with Brefeldin A for 3 h (*n* = 3). **(I)** Percentage of TNFα^+^ cells in hepatic Ly6C^low^ macrophages in wild-type mice after in vitro culture with lipid A (1 µg/ml) and Brefeldin A for 3 h, with or without LXR agonist (*n* = 3). **(J)** Survival curve of *Rt2*^*−/−*^ mice challenged with APAP at 750 mg/kg. Indicated mice had been intravenously administered with clodronate at 25 mg/kg 24 h before APAP challenge (*n* > 4). **(K)** Survival curve of wild-type mice left untreated or administered twice with recombinant AIM (400 µg) before APAP challenge (*n* = 10–15). **(L)** Survival curve of wild-type mice left untreated or administered twice with LXR agonist before APAP challenge (*n* = 5–10). **(M)** Serum levels of AST and ALT in *Rt2*^*−/−*^ mice with or without antibiotic treatment (*n* = 5). **(N)** Survival curve of wild-type mice left untreated or administered twice with the TLR13 ligand Sa19 before APAP challenge (*n* = 5–8). *P < 0.05, **P < 0.01, ***P < 0.001 and ****P < 0.0001.

**Figure S5.**
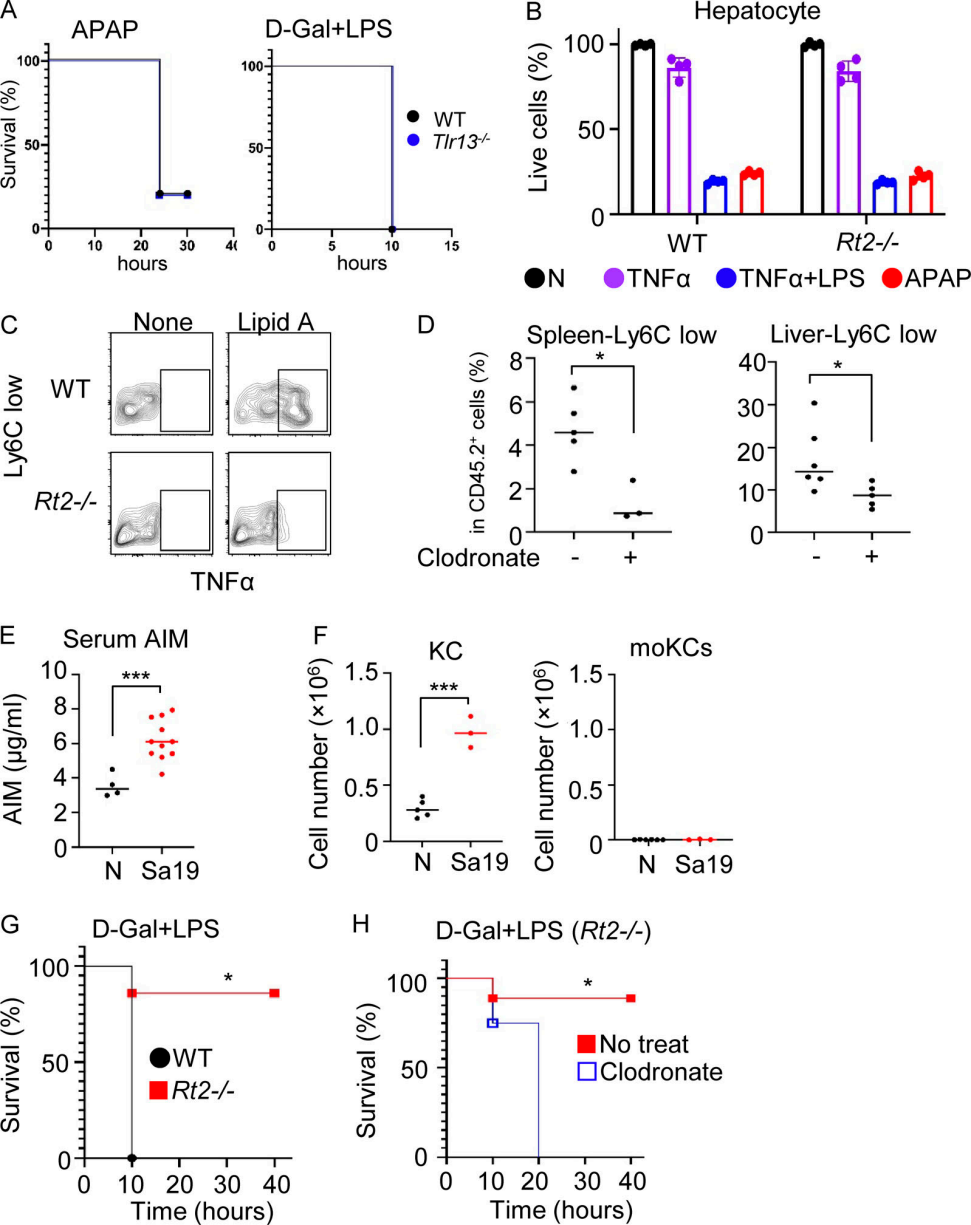
**TLR13 protects against acute liver damage. (A)** Survival curve of indicated mice after APAP challenge at 750 mg/kg or intraperitoneal administration of LPS and D-Gal at 100 ng/mouse and 12.5 mg/mouse, respectively (*n* = 5–10). **(B)** The percentage of live hepatocytes from wild-type and *Rnaset2*^−/−^ mice after stimulation with TNF-α, TNF-α + LPS, or APAP. **(C)** Representative contour plot showing TNF-α^+^ cells in hepatic Ly6C^low^ macrophages from wild-type and *Rnaset2*^*−/−*^ mice after in vitro stimulation with the TLR4 ligand lipid A (1 µg/ml) and Brefeldin A for 3 h. **(D)** Dot plots show the percentage of splenic and hepatic Ly6C^low^ macrophages in wild-type mice treated with clodronate (25 mg/mice) at 16 h prior to analysis (*n* = 4–6). **(E)** Serum levels of AIM in wild-type mice with or without Sa19 administration (*n* = 4–11). **(F)** The numbers of KCs and moKCs in the liver of wild-type mice with and without Sa19 administration. **(G and H)** Survival curve of mice after intraperitoneal administration of LPS and D-Gal at 100 ng/mouse and 12.5 mg/mouse, respectively. Indicated mice intravenously received clodronate at 25 mg/kg 24 h before administration of LPS and D-Gal (*n* = 5–10). *P < 0.05 and ***P < 0.001.

Given that KCs have protective roles against APAP-induced liver injury (Ju et al., 2002), we hypothesized that moKCs accumulated in *Rnaset2*^−/−^ mice also play protective roles in the liver. To test this, *Rnaset2*^−/−^ and wild-type mice were intraperitoneally administered with APAP at a dose of 750 mg/kg. Approximately, 70% of wild-type mice died within 24 h after APAP administration (Fig. 7 A), whereas all *Rnaset2*^−/−^ mice survived the APAP challenge. *Rnaset2*^−/−^*Tlr13*^−/−^ and *Tlr13*^−/−^ mice were as sensitive to the APAP challenge as wild-type mice (Fig. 7, A and B; and Fig. S5 A). APAP causes initial hepatocyte damage, which results in the release of danger signals, macrophage-mediated inflammatory responses, leading to acute liver injury (Imaeda et al., 2009). Hepatocytes from *Rnaset2*^−/−^ mice were as sensitive to the toxic effects of APAP as wild-type hepatocytes in in vitro culture (Fig. S5 B), and elevation of serum levels of alanine aminotransferase (ALT) and aspartate aminotransferase (AST) at 6 h after the APAP challenge were observed in both wild-type and *Rnaset2*^−/−^ mice (Fig. 7, C and D), suggesting that initial hepatocyte damage occurred in *Rnaset2*^−/−^ mice. However, the serum levels of AST and ALT at 24 h after the APAP challenge were still elevated in wild-type mice but not in *Rnaset2*^−/−^ mice. Additionally, neutrophil infiltration into the liver and elevation of serum levels of cytokines such as the neutrophil-attracting chemokine CXCL2 and IL-6 were observed only in wild-type mice (Fig. 7, E–G), suggesting that macrophages could not respond to danger signals from damaged hepatocytes. Given that TLR4 and TLR9 are implicated in APAP-triggered liver injuries (Arai et al., 2016; Maehara et al., 2021), we examined the responses of these TLRs in hepatic macrophages (Fig. 7 H and Fig. S5 C). In wild-type mice, both Ly6C^hi^ and Ly6C^low^ macrophages produced TNF-α in response to lipid A or CpG-B, whereas KCs produced much lower levels of TNF-α. In *Rnaset2*^−/−^ mice, TNF-α production in Ly6C^low^ macrophages (moKCs) was reduced to the levels observed in KCs. Since moKCs predominantly accumulated in the liver of *Rnaset2*^−/−^ mice (Fig. 4 B), impaired inflammatory responses in the liver can be attributed to the impaired TLR responses of moKCs. LXR was activated in wild-type KCs and *Rnaset2*^−/−^ moKCs but not in *Rnaset2*^−/−^ Ly6C^hi^ macrophages (Fig. 5 F), indicating a positive correlation between LXR activation and impaired TLR responses. Consistent with this, the LXR agonist inhibited lipid A–dependent TNFα production by hepatic Ly6C^low^ macrophages from wild-type mice (Fig. 7 I). These results suggest that *Rnaset2*^−/−^ mice survived the APAP challenge in part due to impaired inflammatory responses in moKCs.

In addition to impaired inflammatory responses, we suspected that moKCs play a protective role against the APAP challenge because macrophage depletion using clodronate rendered *Rnaset2*^−/−^ mice susceptible to the APAP challenge (Fig. 7 J and Fig. S5 D). Upregulated genes in moKCs included tissue clearance genes such as AXL and MerTK, both of which play protective roles against acute liver injuries (Ishida et al., 2006; Triantafyllou et al., 2018). In addition, we suspected the protective role of the LXR target gene AIM because it protects against tissue injuries in the kidney and brain by promoting the clearance of danger signals (Akashi-Takamura et al., 2006; Leist et al., 1995). Administration of AIM to wild-type mice before the APAP challenge provided protection against acute liver injury, similar to the effect of the LXR agonist (Fig. 7, K and L). These results suggest that tissue clearance genes upregulated in moKCs, such as AXL, MerTK, and AIM, protected against the APAP challenge (Zagórska et al., 2020).

We next investigated the role of microbiota-derived rRNAs in the protection. Antibiotics treatment rendered *Rnaset2*^−/−^ mice sensitive to the APAP challenge, as indicated by the increased levels of serum AST and ALT (Fig. 7 M). Additionally, administering the TLR13 ligand Sa19 to wild-type mice significantly enhanced their resistance to the APAP challenge (Fig. 7 N). These results suggest that microbiota-derived rRNAs confer protection against acute liver injury. Sa19 administration increased serum levels of AIM and the numbers of KCs but not moKCs (Fig. S5, E and F). The maturation of moKCs depends on the liver niche (Bonnardel et al., 2019; Sakai et al., 2019), but that is occupied by KCs in wild-type mice. Sa19-mediated increases in KCs indicate that TLR13-dependent increases in KCs/moKCs area dependent on the liver niche.

Lastly, we examined responses to LPS and D-Gal, which cause acute liver failure through TNF-α-dependent hepatocyte apoptosis (Akashi-Takamura et al., 2006; Leist et al., 1995). All wild-type and *Tlr13*^−/−^ mice died within 20 h after the LPS/D-Gal challenge, whereas over 80% of *Rnaset2*^−/−^ mice survived (Fig. S5, A and G). Hepatocytes from *Rnaset2*^−/−^ mice were as sensitive to the toxicity of TNF-α and LPS as those from wild-type mice (Fig. S5 B). Similar to the APAP challenge, macrophage depletion by clodronate rendered *Rnaset2*^−/−^ mice susceptible to the LPS/D-Gal challenge (Fig. S5 H). These results suggest that moKCs protected *Rnaset2*^−/−^ mice against acute liver injuries by impairing innate immune responses and upregulating the expression of tissue clearance genes such as AXL, MerTK, and AIM.

## Discussion

We here show that RNase T2 deficiency initiates macrophage TLR13 responses to microbiota-derived rRNAs, leading to macrophage accumulation in the spleen and liver. Taken together with the report by Gomez-Diaz et al. (2025), *Rnaset2*^−/−^ mice have revealed that RNase T2 negatively regulates TLR13 responses by degrading its ligand, bacterial rRNAs.

Constitutive TLR13 activation caused macrophage accumulation by promoting macrophage replenishment. While the roles of the TLR family of receptors in initiating defense responses through the production of proinflammatory cytokines and type I IFNs are well-documented (Kaisho and Akira, 2006; Miyake et al., 2018), the involvement of TLRs in macrophage replenishment remains less well understood. Macrophages residing in peripheral organs are classified into two types: moMAs and tissue-resident macrophages (Ginhoux and Guilliams, 2016). During infections, TLR ligands enhance monocyte production in the BM and spleen through myeloid-biased and extramedullary hematopoiesis, a process known as emergency myelopoiesis (Burberry et al., 2014; Chavakis et al., 2019; Nagai et al., 2006; Ueda et al., 2005). This mechanism replenishes the moMA pool in peripheral organs to bolster defense responses. Increases in monocyte progenitors in the spleen of *Rnaset2*^−/−^ mice indicate that lysosomal ssRNAs activate emergency myelopoiesis, thereby expanding the macrophage pools of the spleen and liver.

Splenic macrophages, increased by emergency myelopoiesis, continued to be activated by TLR13, leading to their proliferation and subsequent production of IL-10 but not proinflammatory cytokines. Tissue macrophages that produce IL-10 upon TLR ligand stimulation have been reported in other organs such as the intestine (Sakai et al., 2019; Scott et al., 2016), where they play immunoregulatory roles. Intestinal macrophages produce IL-10 in a manner dependent on commensal microbiota (Shibata et al., 2023). TLR13 response to bacterial rRNAs may contribute to the replenishment of IL-10–producing macrophages in the intestine as well as in the spleen. As IL-10 is an anti-inflammatory, tissue-protective cytokine (Saraiva et al., 2020), IL-10–producing macrophages in the spleen might contribute to protection against tissue damage in the spleen.

In the liver, TLR13 drove the proliferation of Ly6C^hi^ macrophages and their differentiation into moKCs instead of IL-10–producing macrophages. A comparison of splenic and moKCs revealed stronger LXR activation in moKCs. This difference might be ascribed to LXR agonists, which are steadily produced by hepatocytes to initiate and maintain KCs and moKCs (Sakai et al., 2019). In wild-type mice, Sa19 administration increased KCs, not moKCs, likely because KCs already occupied the liver niche and consumed LXR agonists. In *Rnaset2*^−/−^ mice, monocyte-derived hepatic macrophages likely occupy the niche shortly after birth when the niches are still open to circulating monocytes (Scott et al., 2016). Despite the lack of liver niche signals, TLR13 in splenic Ly6C^low^ macrophages were able to induce expression of LXR target genes such as LXRα and AIM, albeit much weaker than in the liver. Additionally, the TLR13 ligand Sa19 activated LXR in the J774 macrophage line. These results suggest that TLR13-dependent LXR activation works in concert with the hepatic niche signals to induce monocyte/macrophage maturation into moKCs.

LXR activation in accumulated moKCs contributed to protection against acute liver injury by inhibiting inflammatory responses while promoting the expression of tissue clearance genes such as AIM, MerTK, and Axl. AIM-expressing macrophages accumulated also in the lung of *Rnaset2*^−/−^ mice, suggesting that accumulated lung Ly6C^low^ macrophages also have a role in tissue clearance. A lung-specific signal, like LXR ligands in the liver, might work together with TLR13 to drive monocyte/macrophage maturation into LXR-activated macrophages. These results suggest that tissue-specific signals from the niches impact the maturation of moMAs.

Bacterial 23S rRNAs were detected in the vasculature of wild-type and *Rnaset2*^−/−^ mice, indicating that bacterial rRNAs steadily enter into the circulation from the gut. This may not be surprising because the majority of small RNAs on human plasma lipoproteins are derived from bacterial sources, specifically rRNA-derived small RNAs (Allen et al., 2018). KCs and other tissue macrophages continuously degrade bacterial rRNAs by RNase T2. In the assay system using Ba/F3 cells, RNase T2 deficiency enhanced TLR13 responses to ssRNAs of varying length from 19 nt to 3 kb, demonstrating that, like TLR3 (Liu et al., 2021), TLR13 is negatively regulated by RNase T2. Despite the accumulation of ssRNA in lysosomes, TLR7 deficiency did not ameliorate splenomegaly and hepatomegaly in *Rnaset2*^−/−^ mice, likely because TLR7 responses are impaired in *Rnaset2*^−/−^ mice (Greulich et al., 2019; Liu et al., 2021). In wild-type mice, both TLR13 and TLR7 would respond to bacterial 23S rRNAs. Given that hepatomegaly did not develop in *Slc29a3*^−/−^ mice (Shibata et al., 2023), it is possible that TLR7 responses in hepatic macrophages differ from TLR13 responses. In humans, TLR7 and TLR8, instead of TLR7 and TLR13, respond to ssRNAs. Because macrophage TLR8 responds to LDL-derived microbial RNAs (Allen et al., 2022), TLR8 might promote the replenishment of KC in humans.

Production of autoantibodies to RNA-associated autoantigens was triggered in *Rnaset2*^−/−^ mice. As B and T cells do not express TLR13, cDCs and macrophages are likely to drive autoantibody production through the activation of T and B cells. A gut pathobiont, *Enterococcus gallinarum*, translocates to the liver and triggers autoimmune responses in humans and mice (Manfredo Vieira et al., 2018). TLR8 and TLR13 in hepatic DCs and macrophages might promote the presentation of RNA-associated autoantigens to T cells in the liver in humans and mice, respectively.

Although our *Rnaset2*^−/−^ mice were consistent with the previous report in the phenotypes such as splenomegaly, hepatomegaly, macrocytic anemia, thrombocytopenia, and production of autoantibodies to RNA-associated antigens, some phenotypes reported previously were not observed in our *Rnaset2*^−/−^ mice. *Rnaset2*^−/−^ mice in the previous report died earlier than wild-type mice but our *Rnaset2*^−/−^ mice did not show premature death even at 50 wk of age. Although T cells were increased in the liver of our *Rnaset2*^−/−^ mice, serum levels of AST and ALT did not increase, demonstrating that hepatocytes were not damaged at the unperturbed state. The lack of these pathologies might be explained by the lack of increases in expressions of mRNAs for type I IFN and ISGs in splenic and hepatic macrophages of our *Rnaset2*^−/−^ mice. It may be important to additionally study expressions of type I IFNs and ISGs in other organs such as the brain. Because TLR13 responded to microbiota-derived bacterial 23S rRNA, microbiota might be distinct between *Rnaset2*^−/−^ mice in the previous report and this study. Alternatively, TLR13-independent innate immune responses are strongly activated in *Rnaset2*^−/−^ mice of the previous report. Activation of cytoplasmic RNA sensors such as RIG-I and MDA-5 might cause type I IFN-dependent pathologies in *Rnaset2*^−/−^ mice.

In conclusion, we here show that TLR13 responses to bacterial 23S rRNAs activate monocyte progenitors to trigger emergency myelopoiesis, and macrophages to proliferate and mature into anti-inflammatory and tissue clearance macrophages such as IL-10–producing macrophages and moKCs in the spleen and liver, respectively. ssRNAs might serve as an environmental cue to promote tissue clearance through the replenishment of macrophages in mice.

## Materials and methods

### Mice

C57BL/6 mice (sex: male and female; weight: 14–20 g) were purchased from Japan SLC, Inc. *Rnaset2a*^−/−^*Rnaset2b*^−/−^ and *Unc93b1*^−/−^ mice were previously described (Fukui et al., 2018; Liu et al., 2021). In this manuscript, we refer to *Rnaset2a*^−/−^*Rnaset2b*^−/−^ mice as *Rnaset2*^−/−^ mice for simplicity. C57BL/6 *Tlr3*^−/−^ and *Tlr7*^−/−^ mice were kindly provided by Professor Shizuo Akira (Osaka University, Osaka, Japan) and have been previously described (Hemmi et al., 2002; Yamamoto et al., 2003). All the animals were housed in specific pathogen–free facilities at the Institute of Medical Science, University of Tokyo (IMSUT). All animal experiments were approved by the Institutional Animal Care and Use Committee of the IMSUT (#PA17-84, #PA22-43).

### Generation of *Tlr13*^−/−^ mice

CMTI-2 (Bruce4) Embryonic Stem (ES) cells were transfected with the vectors targeting the *Tlr13* locus (Fig. S2 A), and clones resistant to G418 and ganciclovir were screened for homologous recombination using PCR and confirmed using Southern blot analysis. Targeted ES clones were injected into BALB/c-derived blastocysts to generate chimeric mice, which were mated to obtain *Tlr13*^−/−^ mice. *Tlr13*^−/−^ mice were typed by PCR using primers (Primer#1: 5′-TCG​GAA​ACC​TAC​CCA​AGT​TAG​AGA​CAC-3′, Primer#2: 5′-TAA​CTC​CTG​CAA​ACT​ACC​CAA​TCC​TTG-3′, Primer#3: 5′-ATC​GCC​TTC​TAT​CGC​CTT​CTT​GAC​GAG-3′).

### Generation of anti-mouse Tlr13 mAb

To establish an anti-mouse TLR13 mAb, *Tlr13*^−/−^ mice were immunized several times with Ba/F3 cells expressing HA tag-conjugated mouse TLR13 and CFP-conjugated UNC93B1. 5 days after the final immunization, splenic cells were fused with Sp2/o-Ag myeloma cells using polyethylene glycol (PEG1500; Roche). After hypoxanthine–aminopterin–thymidine (HAT supplement; Gibco) selection, a hybridoma producing anti-mouse TLR13 mAb was selected by staining of Ba/F3 cells expressing mTLR13-HA and parental Ba/F3 cells. Finally, the M13E7 clone (anti-mouse TLR13, mouse IgG1/κ) was established by limiting dilution. Biotinylated and PE-conjugated mAb were prepared using Biotin Labeling Kit NH_2_ and R-Phycoerythrin Labeling Kit (Dojindo), respectively.

### Reagents

The LXR agonist T0901317 and LXR antagonist GSK2033 were purchased from Selleck Chemicals, clodronate from Funakoshi, and APAP from TCI Chemicals. The EdU used in the in vitro proliferation assay was purchased from Tokyo Chemical Industry Co. Lipid A purified from *Salmonella minnesota* (Re-595) and lipopolysaccharide (LPS) from *Escherichia coli* (O55:B5) were purchased from Sigma-Aldrich (Merck). Pam3CSK4, poly(I:C), and R848 were purchased from InvivoGen. Sa19 (19mer, GsGsAsCsGsGsAsAsAsGsAsCsCsCsCsGsUsGsG) and CpGB ODN1668 (dTsdCsdCsdAsdTsdGsdAsdCsdGsdTsdTsdCsdCsTdsdGsdAsdTsdGsdCsdT), in which “s” depicts a phosphorothioate linkage, were synthesized by FASMAC. Recombinant AIM preparation has been described previously (Maehara et al., 2021). Lipofectamine 2000 was purchased from Invitrogen (Thermo Fisher Scientific) and DOTAP from Sigma-Aldrich.

### Antibodies

Rat anti-mouse TLR1 monoclonal antibody (mAb) (TR23), rat anti-mouse TLR2 mAb (CB225), mouse anti-mouse TLR5 mAb (ACT5), mouse anti-mouse TLR6 mAb (C1N2), mouse anti-mouse TLR3 mAb (PaT3), mouse anti-mouse TLR7 mAb (A94B10), and mouse anti-mouse TLR9 mAb (J15A7) were established in our laboratory. Phycoerythrin (PE)-conjugated mouse anti-TLR3 mAb (PaT3), PE-conjugated mouse anti-TLR7 mAb (A94B10), and PE-conjugated mouse anti-TLR9 mAb (J15A7) were purchased from BD Biosciences. Biotinylated mAbs were prepared using Biotin-XX (Thermo Fisher Scientific). Biotinylated mouse anti-mouse TLR4 mAb (UT49) was provided by Dr. Hiroki Tsukamoto (International University of Health and Wefare, Fukuoka, Japan). PE rat IgG2a isotype control antibody and PE mouse IgG2b-d κ isotype control antibody were purchased from BioLegend, and PE mouse IgG1-κ isotype control antibody from BD Biosciences. Monoclonal anti-mouse CD11b (clone M1/70), CX3CR1 (clone SA011F11), F4/80 (clone BM8), NK1.1 (clone PK136), CD16.2 (clone 9E9), CD3ε (clone 145-2c11), CD19 (clone 6D5), CD11c (clone N418), CD317 (clone 927), CD45.2 (clone 104), CD8 (clone 53-6.7), Ki67 (clone 16A8), Tim4 (clone F31-5G3), CD34 (clone HM34), CD16/32 (clone 93), CD135 (clone A2/F10), and Sca-1 (clone D7) antibodies were purchased from BioLegend.

Monoclonal anti-mouse CD49b (clone Hmα2), IA/IE (clone M5/114.15.2), Ly6C (clone HK1.4), and Ly6G (clone 1A8) CD115 (clone T38-320), CD117(clone2B8), TER-119 (clone TER-119) antibodies were purchased from BD Biosciences. Monoclonal anti-mouse CD4 (clone RM4-5) antibody was purchased from Invitrogen and monoclonal anti-mouse Axl antibody (clone 175128) from R&D Systems. Polyclonal anti-mouse LXRα antibody (#ab3585) was purchased from Abcam. The rabbit anti-mouse AIM antibody (clone rab1) was provided by Dr. Miyazaki (Tokyo, Japan). The LEGENDScreen Mouse PE Kit was purchased from BioLegend.

### Cell preparation

Blood cells were obtained from mice, using a microtube with EDTA (Erma Inc.). Spleens were minced using glass slides and pipetted several times to disperse the cells in RPMI 1640 medium. The suspended samples were teased using nylon mesh to remove tissue debris. Livers were minced and processed using a gentle MACS Octo Dissociator with Heaters (Miltenyi Biotec). Supernatants were filtered using MACS SmartStrainer (pore size: 100 µM; Miltenyi Biotec) and centrifuged at 300 × *g* for 10 min. The pellet was resuspended in Debris Removal Solution (Miltenyi Biotec) and centrifuged at 3,000 × *g* for 10 min. All cell pellet was resuspended in RBC lysis buffer (BioLegend).

### Flow cytometry

Cell surface staining for flow cytometric analysis was performed using fluorescence-activated cell sorting (FACS) staining buffer (1× phosphate-buffered saline [PBS] with 2.5% fetal bovine serum and 0.1% NaN_3_). The prepared cell samples were incubated for 10 min with an unconjugated anti-mouse CD16/32 blocking mAb (clone 95) to prevent nonspecific staining in the staining buffer. The cell samples were then stained with fluorescein-conjugated monoclonal antibodies for 20 min on ice. Stained cells were fixed with BD Cytofix Fixation Buffer (BD Biosciences) for 20 min at 4°C and washed with the staining buffer. For intracellular staining of TLR3, 7, and 9, fixed cells were permeabilized using BD Perm/Wash buffer (BD Biosciences) and incubated with anti-TLR antibody or isotype control IgG1 for 30 min at 4°C. For intracellular staining of mouse IL-6 and TNF-α in cells from spleen and liver stimulated with various ligands in the presence of Brefeldin A (10 µg/ml), cell surface stained cells were fixed and permeabilized using BD Perm/Wash buffer and incubated with anti-mouse IL-6 mAb (clone MP5-20F3; BioLegend) or anti-mouse TNF-α mAb (clone MP6-XT22; BioLegend) antibody or isotype control IgG for 1 h at 4°C. For intracellular staining of AIM, cell-surface stained cells were fixed and permeabilized using True-Nuclear Transcription Factor Buffer Set (BioLegend), incubated with anti-AIM antibody for 30 min at 4°C, and washed with True-Nuclear Perm Buffer. The stained cells were incubated with PE-conjugated anti-rabbit IgG for 30 min at 4°C and washed with True-Nuclear Perm Buffer. The stained cells were analyzed using an ID7000 spectral cell analyzer (Sony Biotechnology). All data were analyzed using FlowJo software (BD Biosciences).

### Phosphorylation of signaling molecules was detected by flow cytometry

Cell surface stained cells were fixed with Cyto-Fast Fix/Perm buffer (BioLegend) for 20 min at room temperature and washed twice with FACS staining buffer. Fixed cells were further permeabilized by adding prechilled True-Phos Perm Buffer (BioLegend) and incubated at −20°C for 2–3 h. After washing twice with FACS staining buffer, permeabilized cells were stained with PE-conjugated anti-p-Syk (clone C87C1; 1:50 dilution; Cell Signaling Technology), anti-p-S6 (clone D57.2.2E; 1:100 dilution; Cell Signaling Technology), anti-p-GSK3β (clone D85E12; 1:50 dilution; Cell Signaling Technology), anti-p44/42 MAPK (Erk1/2; clone 137F5; 1:50 dilution; Cell Signaling Technology), or Fluor647-conjugated anti-active β-catenin (clone 8E7; 1:400 dilution; Merck Millipore) and subjected to flow cytometry analyses.

### Cell sorting

Cell sorting was conducted using the FACS ARIA III Cell Sorter (BD Biosciences). To purify Ly6C^lo^ and Ly6C^high^ splenic monocytes, splenocytes from wild-type and Rnaset2^−/−^ mice were incubated with biotinylated anti-mouse CD3 (clone 145-2C11)/CD19 (clone 6D5)/NK1.1 (clone PK136)/Ly6G (clone aA8)/TER-119/erythroid cells (clone Ter-119), followed by incubation with Streptavidin MicroBeads (Miltenyi Biotec). The magnetically labeled cells were removed using autoMACS (Miltenyi Biotec), and the enriched cells were stained with anti-mouse CD45.2, F4/80, CD11b, Ly6C, CD16.2, NK1.1, and Ly6G mAbs. Ly6C^lo^ CD16.2^high^ and Ly6C^high^ CD16.2^lo^ CD11b^+^NK1.1^−^Ly6G^−^ cell populations were sorted. For sorting of KCs and hepatic macrophages from wild-type mice and Ly6C^lo^ and Ly6C^high^ hepatic macrophages from Rnaset2^−/−^ mice, liver cells were stained with antibodies against CD45.2, F4/80, CD317, CD11b, Ly6C, and CD16.2. F4/80^+^ CD11b^lo^, F4/80^+^ CD11b^hi^, F4/80^+^ CD11b^lo^ CD317^hi^ CD16.2^lo^, and F4/80^+^ CD11b^lo^ CD317^lo^ CD16.2^hi^ cells were sorted as KCs, hepatic macrophages, AIM^lo^ KCS, and AIM^high^ KCs, respectively. F4/80^+^ CD11b^hi^ Ly6C^lo^ CD16.2^hi^ and F4/80^+^ CD11b^hi^ Ly6C^lo^ CD16.2^hi^ cells in *Rnaset2*^−/−^ mice were sorted as Ly6C^lo^ hepatic macrophages and Ly6C^high^ hepatic macrophages.

### RNAseq analysis

Total RNA was extracted from sorted cells using RNeasy Mini Kits (Qiagen), and the quality of the RNA was evaluated using an Agilent Bioanalyzer (Agilent Technologies). Samples with an RNA integrity number value >7.0 were subjected to library preparation. RNA-seq libraries were prepared with 1 ng of total RNA using the Ion AmpliSeq Transcriptome Mouse Gene Expression kit (Thermo Fisher Scientific) according to the manufacturer’s instructions. The libraries were sequenced with 100-bp single-end reads to a depth of at least 10 million reads per sample on the Ion Proton platform, using an Ion PI Hi-Q Sequencing 200 kit and Ion PI Chip v3 (Thermo Fisher Scientific). The FASTQ files were generated using AmpliSeqRNA plug-in v5.2.0.3 in the Torrent Suite software (v5.2.2; Thermo Fisher Scientific) and analyzed using the TCC-GUI software. Individual sample reads were normalized to the relative log expression using the DESeq2 R library. DESeq2 was used to determine the fold changes and P values. Genes showing >1.5-fold change in expression (adjust P < 0.05) were considered significantly altered. To interpret the gene expression profiles, GSEA was performed using GSEA 4.1.0, with the MSigDB hallmark gene sets. Enriched pathways were determined by false discovery rate (FDR)-adjusted P values <0.1 To identify the activation transcription factors, overrepresentation analysis was conducted using Enrich R (https://maayanlab.cloud/Enrichr/) with ARCHS4 TFs Coexp.

### Proliferation assay with EdU labeling

In vitro proliferation assays were conducted using the Click-iT Plus EdU Alexa Fluor 488 Flow Cytometry Assay Kit (Invitrogen), according to the manufacturer’s instructions. Spleen, liver, and blood samples were collected from the mice. Erythrocytes were then completely lysed using BD Pharm Lyse lysing buffer (BD Biosciences) to collect splenocytes and peripheral blood mononuclear cells (PBMCs). Collected cells were incubated with 1 µg/ml EdU for 1 h. After blocking splenocytes and PBMCs with an anti-CD16/32 (clone 95) mAb, the samples were stained with fluorescent dye–conjugated mAbs. The stained samples were subsequently fixed with BD Cytofix (BD Biosciences) and permeabilized using 1× Click-iT saponin-based permeabilization and washing reagents. Finally, EdU incorporated into the genomic DNA was stained using the Click-iT EdU reaction cocktail. EdU-positive cells were detected using the abovementioned spectral flow cytometer ID7000 (Sony Biotechnology).

### In vitro cell proliferation assay

Whole mouse splenocytes were plated at a density of 2 × 10^6^ cells per well in a Cepallet W-type 24-well microplate (DIC) and cultured for 3 days with or without mouse/human M-CSF (Peprotech). Surviving macrophages that adhered to 24-well plates were detached by lowering the temperature on ice. The collected cells were counted using an automated cell counter CellDrop BF (DeNovix). Cells were stained with CD11b/Ly6G/NK1.1/Ly6C/Fcgr4 after blocking antibody treatment and analyzed using an ID7000 spectral cell analyzer.

### RNA extraction of lysosome isolation

Lysosomes were isolated from spleen and liver cells using Lysosome Enrichment Kit for tissues and cultured cells (Thermo Fisher Scientific). RNA was extracted from lysosomes using TRIzol (Thermo Fisher Scientific), and the RNA quantity was measured using a Qubit Fluorometer (Thermo Fisher Scientific).

### Histological analysis

Mouse tissues were fixed in a 20% formalin neutral buffer solution. Fixed kidneys were embedded in paraffin wax for sectioning. Sections were subjected to hematoxylin and eosin (HE) staining or immunohistochemistry for F4/80 and AIM and visualized using an EVOS microscope (Thermo Fisher Scientific).

### Biochemical test

Sera were collected from mice aged 30–45 wk. AST and ALT levels were measured using the Biochemical automatic analyzer JCA-BM6050 (JEOL Ltd.) in ORIENTAL YEAST Co., Ltd.

### Platelet and cell count

Platelet numbers in PBMCs were analyzed using an automatic hematology analyzer Celltac α (Nihon Kohden), and cells were counted using an automated cell counter CellDrop BF.

### TLR ligand injection

Each ligand was diluted in PBS. Mice were intraperitoneally administered with Sa19 (2 µg/mice with Lipofectamine) for two consecutive days.

### BM transfer

C57BL/6 WT CD45.1 mice at 6–7 wk of age were lethally irradiated at 4.75 × 2 Gy using MBR-1520R-4 (Hitachi Power Solutions) and received 1 × 10^6^ BM cells from *Rnaset2*^*−/−*^ mice through intravenous routes.

### Establishment of APAP-induced mouse liver injury

Mice were allowed free access to water but not food for 16 h before the APAP challenge. APAP was dissolved in PBS containing 10% DMSO. In preliminary experiments, mice were intraperitoneally administered with an APAP solution at a dose of 250–750 mg/kg. To determine the survival rate, a dose of 750 mg/kg was administered. To evaluate liver injury based on serum AST and ALT levels and neutrophil infiltration into the liver, we selected 500 mg/kg as the APAP dose. For experimental intervention, mice were intravenously administered with rAIM (400 µg/mice) at the same time as the APAP challenge; clodronate (25 mg/mice) at 16 h before the APAP challenge; or Ly6C^lo^ hepatic macrophages from *Rnaset2*^−/−^ mice (1 × 10^6^ cells/mice) 16 h before the APAP challenge.

### Lentivirus transduction

To establish mutant cells, the LCV2-gRNA vector (Rnase t2 gRNA: 5′-CCG​GGC​TGG​ATC​TCC​GTG​C-3′) was transfected into the HEK293FT cells with ViraPower Lentiviral Expression Systems (Invitrogen). After 2 days of incubation, the supernatants were obtained as viral suspensions. Ba/F3 cells were infected by the viral suspensions.

### Retrovirus transduction

TLR3, TLR7, TLR13-HA tag, and Unc93B1 were amplified by PCR from mouse genomic DNA. All cDNAs were cloned into retroviral pMXpuro vectors provided by T. Kitamura. The NEBuilder HiFi DNA Assembly Cloning Kit (New England BioLabs) and Rapid DNA Ligation Kit (Roche Applied Science) were used for the cloning. The pMXpuro vectors were transfected into Plat-E packaging cells with FuGene6 (Roche Applied Science). After 1 day of incubation, the supernatants were obtained as viral suspensions. Ba/F3 cells expressing NFκB GFP reporter plasmid were infected by the viral suspensions mixed with DOTAP (Roche Applied Science).

### Preparation of TLR13 ligand

Each TLR13 ligand was amplified by PCR from mouse fecal DNA extracted with NucleoSpin DNA Stool (Takara). 23S-Fw: 5′-CCG​TGT​ACG​CTT​AGT​CGC​TTA-3′, 23S-Rv: 5′-ATG​AAC​CGT​GAG​GCT​TAA​CCT​T-3′, 1684-Fw (for 700 nt): 5′-GTG​CCT​TCT​CCC​GAA​GTT​AC-3′, 2384-Rv (for 700 and 350 nt): 5′-GAG​CAG​GTG​CGA​AAG​CAG​GT-3′, 2035-Fw (for 70 and 350 nt): 5′-TCT​TGC​CGC​GGG​TAC​ACT​GC-3′, 2104-Rv (for 70 nt): 5′-GCT​TGA​CAC​TGA​ACA​TTG​AGC​C-3′.

### ELISA

Anti-Sm and anti-SSA/Ro60 antibodies were quantified using an ELISA kit (Alpha Diagnostic International Inc.). Serum levels of anti-double-stranded DNA antibodies were measured using a commercial ELISA kit (FUJIFILM Wako Pure Chemical Corporation), serum IL-10 and CXCL2 levels were measured using a DuoSet ELISA kit (R&D Systems), and serum IL-6 and TNF-α levels were measured using a commercial ELISA kit (Thermo Fisher Scientific). Serum AIM levels were measured using ELISA, as described previously (Maehara et al., 2021).

### Online supplemental material

Fig. S1 shows the phenotypes of *Rnaset2*^*−/−*^ mice and generation of *Tlr13*^*−/−*^ mice. Fig. S2 shows myeloid progenitors in the spleen and BM, Ki67 expression in splenic macrophages, transcriptome analyses of splenic macrophages, survival/proliferation of splenic macrophages, and the mRNA expression of ISGs in the spleen and liver. Fig. S3 shows FACS analyses, the numbers of macrophages, TLR13 expression, and proliferation of macrophages in the brain, lung, and kidney. FACS analyses, in macrophages from wild-type, *Rnaset2*^*−/−*^, and *Rnaset2*^*−/−*^*Tlr13*^*−/−*^ mice. Fig. S4 shows mRNA expression of LXR and MafB target genes in hepatic macrophages, AIM protein expression in macrophages from the brain, lung, and kidney, LXRα subcellular distribution in the J774 macrophage line, validation of the TLR13 ligand, and LXR activation in J774. Fig. S5 shows mouse survival after challenge with APAP and D-Gal + LPS, hepatocyte survival in vitro with APAP, TNFα production by macrophages stimulated with lipid A, macrophage depletion by clodronate treatment, serum AIM, the numbers of KCs and moKCs after treatment with the TLR13 ligand Sa19, and survival after challenge with D-Gal + LPS. Data S1 shows summarized RNA-seq data of splenic and hepatic macrphages from WT and *Rnaset2*^−/−^ mice.

## Supplementary Material

Data S1 shows summarized RNA-seq data of splenic and hepatic macrphages from WT and *Rnaset2*^−/−^ mice.

SourceData F1 is the source file for Fig. 1.

SourceData FS1 is the source file for Fig. S1.

## Data Availability

All the RNA sequence data analyzed in this manuscript were deposited in the NCBI Gene Expression Omnibus database under accession number GSE281642. All other data are available in the main text or the supplementary material.
