# Supplementary figures and images for: RNase T2 deficiency promotes TLR13-dependent replenishment of tissue-protective Kupffer cells
Source: J Exp Med. 2025 Jan 24;222(3):e20230647. doi: 10.1084/jem.20230647 (PMC11758922; doi:10.1084/jem.20230647)

SourceDataF1E

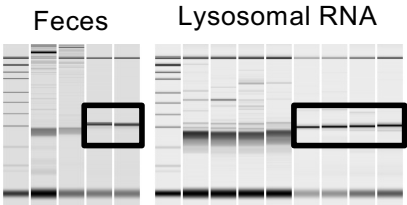

Supplement: SourceData F1 — is the source file for Fig. 1. [file jem_20230647_sourcedataf1.pdf]

SourceDataFS1C

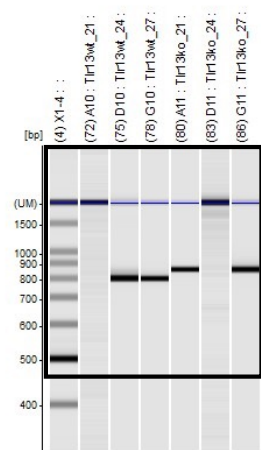

SourceDataFS1G

RNaseT2

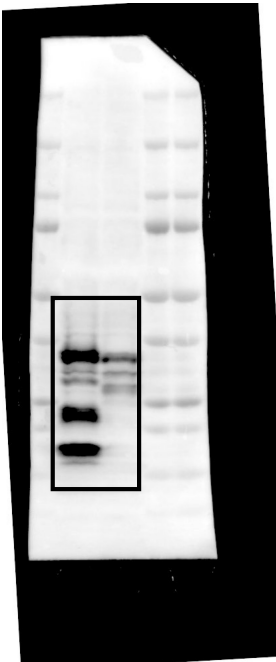

beta-actin

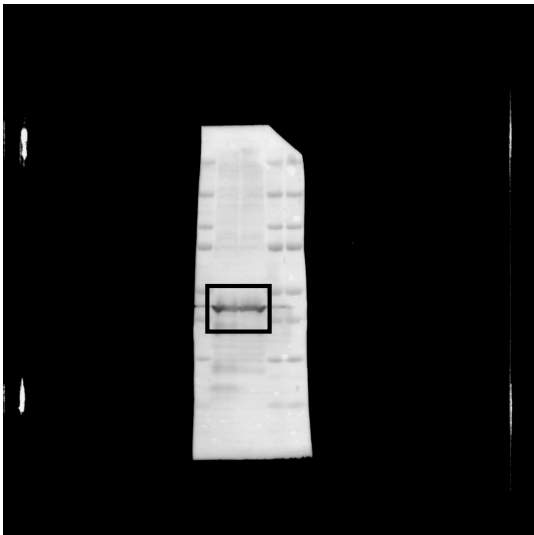

Supplement: SourceData FS1 — is the source file for Fig. S1. [file jem_20230647_sourcedatafs1.pdf]
